# Titin gene mutations enhance radiotherapy efficacy via modulation of tumour immune microenvironment in rectum adenocarcinoma

**DOI:** 10.1002/ctm2.70123

**Published:** 2025-01-02

**Authors:** Hengchang Liu, Jialiang Liu, Xu Guan, Zhixun Zhao, Pu Cheng, Haipeng Chen, Zheng Jiang, Xishan Wang

**Affiliations:** ^1^ Department of Colorectal Surgery National Cancer Center/National Clinical Research Center of Cancer/Cancer Hospital Chinese Academy of Medical Sciences and Peking Union Medical College Beijing China

**Keywords:** ANKRD1, anti‐tumour effects, DNA damage repair, radiotherapy sensitivity, rectum adenocarcinoma, T‐cell infiltration, TTN mutations

## Abstract

**Objective:**

This study investigates the impact of Titin (TTN) gene mutations on radiotherapy sensitivity in rectum adenocarcinoma (READ) by examining changes in the tumour immune microenvironment.

**Methods:**

Data on gene expression and mutations in READ were obtained from The Cancer Genome Atlas (TCGA) and International Cancer Genome Consortium (ICGC) databases. Bioinformatics analysis explored the correlation between TTN mutations and immune cell infiltration. In vitro, lentiviral vectors were used to assess TTN mutations' effects on ANKRD1 expression in two READ cell lines. ANKRD1 was overexpressed, and clonogenic assays evaluated radiotherapy sensitivity. Flow cytometry, immunofluorescence, and comet assays examined mutations' impact on cell cycle, apoptosis, and DNA damage response (DDR). An in vivo mouse model and formalin‐fixed paraffin‐embedded samples from locally advanced rectal cancer (LARC) patients before and after radiotherapy were analyzed, followed by prognostic evaluation.

**Results:**

Bioinformatics revealed that TTN mutations increase radiation sensitivity in LARC by slowing cell proliferation, promoting apoptosis, and reducing DDR. TTN mutations also inhibit ANKRD1 expression via JUN disruption and enhance CD4/CD8 T‐cell infiltration, improving anti‐tumour immunity and outcomes. Observations from the clinical study showed a substantial decline in ANKRD1 expression levels alongside a notable surge in the counts of CD4^+^ and CD8^+^ T cells after undergoing radiotherapy. Patients with TTN mutations, low ANKRD1 expression, and high densities of CD4^+^ and CD8^+^ T cells had longer 3‐year disease‐free survival in READ.

**Conclusion:**

Our findings reveal that TTN mutations can serve as biomarkers for enhanced radiotherapy sensitivity in READ. By altering the tumour's immune microenvironment, these mutations may provide a novel target for personalized radiotherapy strategies, potentially improving therapeutic outcomes in patients with READ.

**Highlights:**

1. The association between TTN mutations and tumour mutation burden, as well as immune cell infiltration in READ, is examined.
2. TTN mutations enhance the radiation sensitivity of READ cells and weaken DNA damage repair in response to radiation.
3. TTN mutations increase the radiation sensitivity of READ cells by inhibiting ANKRD1.
4. The infiltration of CD8^+^ and CD4^+^ T cells induced by TTN mutations is essential for anti‐tumour immunity.
5. TTN mutations serve as a biomarker for the pathological response to preoperative radiotherapy in READ.

## Graphical abstract

(1) TTN knockout results in decreased ANKRD1 expression, impairing DNA damage repair.

(2) Impaired DNA repair increases apoptosis and enhances radiosensitivity in rectal cancer cells.

(3) Radiotherapy in TTN‐deficient cancer cells induces immune cell infiltration, including CD4^+^ and CD8^+^ T cells.

(4) Apoptosis and immune modulation contribute to improved radiotherapy outcomes in rectal cancer.

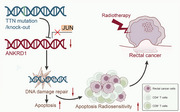

## 1 INTRODUCTION

The global prevalence of rectum adenocarcinoma (READ) as a malignant tumour results in heightened mortality and morbidity rates, constituting a substantial public health challenge.^1^ The complex aetiology and pathophysiology of this disease render its mechanisms not fully understood,^2^ with genetic factors, environmental influences, and lifestyle choices all playing crucial roles.^3^ Dealing with READ typically entails surgery, chemotherapy, and radiotherapy, with radiotherapy playing a crucial role in treating locally advanced rectal cancer (LARC).^4^ Given the substantial variability in patient responses to radiotherapy, uncovering the molecular mechanisms influencing radiotherapy sensitivity holds significant clinical relevance.^5^

With advancements in molecular biology and genomics, increasing research is revealing the close relationship between genetic variations and radiotherapy response.^6^ For instance, mutations in genes like BRCA1, BRCA2, and P53 may compromise the DNA repair capacity of tumour cells, thereby enhancing their sensitivity to radiotherapy.^7^, ^8^, ^9^, ^10^ The TTN (Titin) gene, responsible for encoding muscle elastic protein, plays a crucial role in muscle contraction and relaxation processes.^11^ Recent studies suggest that mutations in the TTN gene might be linked to the emergence of diverse medical conditions, encompassing various categories of malignancies.^12^, ^13^, ^14^ However, the mechanisms by which TTN gene mutations impact the response of tumour patients to radiotherapy remain unclear.^12^ Radiotherapy exerts a dual role in modulating the tumour immune microenvironment (TIME) by activating immune responses and potentially inducing immune suppression.^15^ The effect of TTN mutations on altering the TIME and influencing immune cell infiltration and activation remains to be elucidated. Therefore, this study focuses on investigating the impact of TTN gene mutations on the radiotherapy sensitivity of READ.

This investigation involves the utilization of whole exome sequencing technology to assess the genetic profiles of READ patients, identifying key genes that may affect radiotherapy sensitivity and exploring their mechanisms of action. Specifically, we assess how TTN gene mutations influence DNA damage repair by regulating ANKRD1 expression and their effects on the radiotherapy sensitivity of READ cells. Additionally, we investigate how TTN mutations, by altering the TIME, impact the infiltration of CD4 and CD8 T cells, thereby enhancing the activation of anti‐tumour immunity within the body.

These research findings may hold critical guiding implications for the clinical treatment of READ. Provided that mutations in the TTN gene are confirmed to enhance the response of READ cells to radiotherapy, individualized radiotherapy strategies considering the patient's TTN gene status could be created to enhance treatment effectiveness. Moreover, the potential of TTN gene mutations to activate anti‐tumour immunity by altering the TIME may serve as a novel immunotherapeutic target, paving the way for new treatment strategies for READ.

## 2 MATERIALS AND METHODS

### 2.1 Procuring expression and mutation information for READ from the Cancer Genome Atlas and International Cancer Genome Consortium databases

Obtained from the Cancer Genome Atlas (TCGA) website (https://cancergenome.nih.gov/), the nucleotide variation data of the TCGA‐READ dataset was accessed. Specific attention was given to the “Masked Somatic Mutation” data (*N* = 89). TCGA provided the RNA‐seq data for the TCGA‐READ dataset (READ—Counts data), encompassing all available samples of READ (*N* = 89) as well as normal tissue samples (*N* = 2). Corresponding clinical information was also obtained from TCGA, including data on patient age, gender, TNM staging, histological grading, and overall survival (OS) status. Furthermore, the International Cancer Genome Consortium (ICGC) database (http://dcc.icgc.org/releases/current/Projects) was the source of somatic gene mutation details for American READ samples (*N* = 134) as of 27 November 2019.^16^ Only patients with complete clinical data were included, while those with missing information such as TNM staging, gender, age, and survival status were omitted.

### 2.2 Bioinformatics analysis

R software version 4.2.1 and its relevant packages were utilized for statistical analyses in bioinformatics. The Mutation Annotation Format files from TCGA were analyzed and visualized using the “maftools” package in R. Analysis and visualization of mutation data sourced from the ICGC were carried out utilizing the “GenVisR” package in R. Perl was utilized to extract the top 20 genes with the highest mutation rates from both the TCGA and ICGC databases, followed by intersection analysis using the “venn” function to identify genes with high mutation frequencies. Based on the severity of mutations in these genes, the samples were categorized into wild‐type and mutant groups. The relationship between these intersected genes and tumour mutation burden (TMB) was evaluated and visualized using the “ggpubr” package in R.^16^

### 2.3 TMB calculation

The quantification of TMB is based on the total number of coding errors like base substitutions, insertions, or deletions per million bases in somatic cells. In this study, TMB scores for READ were determined by analyzing the Whole Exome Sequencing data retrieved from the TCGA and ICGC databases. The mutation frequency of each sample was calculated using a Perl script based on the ratio of mutations to the exonic length (38 million bases).^17^

### 2.4 Tumour immune environment in READ samples

The CIBERSORT package was utilized to analyze the distribution of 22 immune cell types in tumour tissues obtained from TCGA and ICGC databases. A significance threshold of *p* < .05 was applied to filter the data, resulting in the immune cell composition matrix for each tumour sample. Tumours with TTN mutations were classified into mutation and wild‐type groups, and the comparison of immune cell populations between the two groups was performed with the assistance of the ‘limma’ package. Visual presentations showcasing the relationship between gene mutations and tumour‐infiltrating immune cells were produced using bar and violin plots with the ‘vioplot’ package in R. By utilizing the TIMER database available at https://cistrome.shinyapps.io/timer/, the infiltration levels of six distinct immune cell categories—CD4^+^ T cells, CD8^+^ T cells, B cells, macrophages, neutrophils, and dendritic cells (DC)—were computed based on different gene sets.^18^

### 2.5 Clinical sample collection

In this study, we retrospectively identified patients with LARC confirmed by biopsy, defined as T3‐4 or N+, who underwent fluoropyrimidine‐based neoadjuvant chemoradiotherapy from January 2015 to January 2019, followed by 50.4 Gy radiotherapy, and subsequently underwent surgical resection within 8−11 weeks. A total of 32 patients met the initial criteria. All patients were blindly selected for genetic tumour profiling without prior knowledge of their genomic status. Standard quality control measures were applied to all samples. Inclusion criteria comprised age between 18 and 80 years, undergoing surgical treatment, histologically confirmed diagnosis of LARC, and no history of prior treatments. Tissue samples from tumours and peripheral blood were gathered and preserved for later analyses. The Kaplan–Meier method and log‐rank test were utilized to estimate disease‐free survival (DFS) in the study. Written informed consent was obtained from all participants. Approval for this study, in accordance with the International Ethical Guidelines for Biomedical Research involving Human Subjects by CIOMS, was granted by the Beijing Hospital Research Ethics Committee (no. NCC2016JZ‐06), with all handling of clinical samples strictly following the Helsinki Declaration.^19^, ^20^

### 2.6 Generation of TTN knockout cells and TTN mutant knockout cells

TTN defective cells were created using CRISPR/Cas9 technology with the following sgRNAs: TTN‐sgRNA1: 5′‐ATGTAAGTCGGAGCTCCAAGTGG‐3′; TTN‐sgRNA2: 5′‐CTGACCGGATCTACTGGTACTGG‐3′. The integration of sgRNAs into the Lenti‐CRISPR v2 vector was facilitated by the inclusion of the Cas9 nuclease gene sourced from *Streptococcus pyogenes* (Hanbio Biotechnology Shanghai). To establish stable TTN‐KO cells, cell transduction was performed using the lentiviral Lenti‐CRISPR v2 vector, followed by selection with 4 µg/mL puromycin (A1113803, Gibco, Thermo Fisher Scientific).

The CRISPR/Cas9 editing system was utilized to introduce the TTN‐R5163H (c.15488G > A) mutation into HCT116 (EDC00018, provided by Guangzhou Editgene Biotechnology Co., Ltd.) or SW837 cells (EDC00116, provided by Guangzhou Editgene Biotechnology Co., Ltd.). The selection of cells involved the use of puromycin at a concentration of 4 µg/mL after transfection with the sgRNA1 plasmid and donor sequence. The surviving cells underwent a process of limiting dilution cloning, and TTN‐R5163H heterozygous and homozygous mutant cells were obtained, designated as wild‐type and homozygous mutant cell lines for HCT116 (HCT116^WT/−^; HCT116^−/MUT^) and wild‐type and heterozygous mutant cell lines for SW837 (SW837^WT/WT^; SW837^WT/MUT^) respectively.^21^

### 2.7 Cell culture

The 293T cells were acquired from the American Type Culture Collection (ACS‐4500) and maintained in DMEM (A1443001, Gibco, Thermo Fisher Scientific (China) Co., Ltd.) supplemented with 10% fetal bovine serum (FBS, 12483020, Gibco, Thermo Fisher Scientific (China) Co., Ltd.). McCoy's 5A medium (16600082, Gibco, Thermo Fisher Scientific (China) Co., Ltd.) containing 10% FBS and 1% penicillin/streptomycin (15140148, Gibco, Thermo Fisher Scientific (China) Co., Ltd.) was used for culturing the paired human READ cell lines HCT116 TTN wild‐type and mutant cells (HCT116^WT/−^; HCT116^−/MUT^). The SW837 TTN wild‐type and mutant cells (SW837^WT/WT^; SW837^WT/MUT^) were provided by Guangzhou Adigene Technology Co., Ltd. and cultured in RPMI1640 medium (A1049101, Gibco, Thermo Fisher Scientific (China) Co., Ltd.) supplemented with 10% FBS and 1% penicillin/streptomycin. The mouse cell lines, CT26 cells (CL‐0071/CM‐0071, Wuhan Procell Life Science & Technology Co., Ltd.) and MC38 cells (CC‐Y2123/CC‐Y2123M, Shanghai Enzyme Research Biotechnology Co., Ltd.), were cultured in company‐specific culture media. Incubation of cells was carried out in a humidified incubator at 37°C with 5% CO_2_. Typically, cells were passaged at least twice before experiments and restricted to a maximum of 20 passages throughout the experimental period. Confirming the identity of the cell lines was accomplished by STR genotyping utilizing the Identifier Kit (Applied Biosystems). Mycoplasma contamination in cell cultures was assessed using the Universal Mycoplasma Detection Kit (30‐1012K, ATCC).^22^, ^23^, ^24^

### 2.8 Gene overexpression in infection

The lentiviral overexpression vector pCDH‐CMV‐MCS‐EF1α‐copGFP (Lv‐overexpression vector, CD511B‐1, System Biosciences, USA) was procured to overexpress genes. The lentiviral vector was transfected into 293T cells using Lipofectamine 3000 reagent (L3000015, Invitrogen). The medium composition was altered to 12 mL with 5% FBS after 20 h. Roughly 48 h later, the viral‐containing supernatant was harvested, passed through a  .45 µm cellulose acetate filter (HAWG04700, MF‐Millipore), and frozen at −80°C.

To generate the overexpressing ANKRD1 cell lines (HCT116^WT/−^; HCT116^−/MUT^) and (SW837^WT/WT^; SW837^WT/MUT^), the viral mixture was applied to cells at 40% confluency for a duration of 8 h. Cells were exposed to an extra 10 µg/mL puromycin after 24 h to facilitate cell selection, and the process of establishing stable transfection cell lines included a 4‐week cell culture phase. The groups were divided as follows: HCT116^WT/−^ + oe‐NC (overexpression negative control); HCT116^WT/−^ + oe‐ANKRD1; HCT116^−/MUT^ + oe‐NC; HCT116^−/MUT^ + oe‐ANKRD1; SW837^WT/WT^ + oe‐NC; SW837^WT/WT^ + oe‐ANKRD1; SW837^WT/MUT^+oe‐NC; SW837^MUT/MUT^ + oe‐ANKRD1.

### 2.9 Analysis of TTN mutations

The amplification of Titin gene exons was achieved via polymerase chain reaction (PCR). Sequencing on capillary electrophoresis was performed directly on the PCR products after purification with a DNA purification kit (D7001, Zymo Research), utilizing the Applied Biosystems 3730 DNA Analyzer (4331247, ThermoFisher).^23^, ^24^

### 2.10 Radiation clonogenic survival assay

Cells in the logarithmic growth phase were cultured and evenly distributed into six‐well plates in triplicate. After 24 h, various doses (0, 2, 4, 6, and 8 Gy) were administered to cells using the Radsource RS2000 biological irradiator (Radsource) operating at 160 kV and 25 mA, delivering a dose rate of around 113 cGy/min. Visible colonies appeared following a 9−12 day incubation period at 37°C. Quantification of colonies was performed post‐staining with crystal violet solution.

The ES‐FUCCI plasmid (derived from the biovectorNTCC plasmid vector cell gene repository centre) was utilized to transfect cells for the cell cycle enrichment clonogenic assay. The gathering of FUCCI‐labeled cells was performed utilizing the BD FACSAria Fusion Sorter (BD Biosciences), with a red filter for the G1 phase and green filters for the S, G2, and M phases. 60 mm tissue culture dishes were used to plate the sorted cells, which were then exposed to radiation doses of 0 and 8 Gy. Following a 10−14 day incubation period, colonies underwent fixation with methanol/acetic acid, staining with  .5% crystal violet, and were subsequently examined using a dissecting microscope to calculate the number of colonies or colony‐forming units with a minimum of 50 cells. Determining the survival fraction involved the following calculation: (number of colonies/plated cells) irradiated/(number of colonies/plated cells) unirradiated.^22^ Three iterations of the experiment were completed.

### 2.11 Neutral comet assay experiment

TTN alleles were exposed to ionizing radiation at doses of 0 and 8 Gy, leading to the generation of DNA double‐strand breaks (DSBs). Subsequently, cells were subjected to alkaline lysis, and conducting the neutral comet assay involved the application of the Trevigen comet assay kit (4253‐096‐ESK, Trevigen) adhering to the guidelines outlined by the manufacturer. Cell imaging was conducted employing a fluorescence microscope (ECLIPSE E800, Nikon). The measurement and quantification of comet tail moments were carried out through the CometScore software (TriTek Corp). Each time point necessitated the counting and imaging of at least 50 cells.^22^

### 2.12 Immunofluorescence detection of cellular protein expression post‐X‐ray irradiation

Treatment with ionizing radiation doses of 0 and 8 Gy was performed on the TTN allele genes within the cells. Twenty‐four hours post‐irradiation, culture of cells was conducted on µ‐slide VI chambered slides (Ibidi). The analysis of nuclear foci involved fixing cells in 4% paraformaldehyde at 4°C for 30 min, and then subjecting them to  .2% Triton X‐100 treatment for 10 min. Subsequently, cells were incubated with anti‐γH2AX antibody (ab2893, 1:100, Abcam) diluted in 3% BSA. In the mitotic mutation assay, cells were stained with an anti‐tubulin antibody (ab52866, 1:500, Abcam). After extensive phosphate‐buffered saline (PBS) washes, cells were stained with Alexa Fluor 488 goat anti‐rabbit IgG (#4412S, 1:2000, Cell Signaling Technology) at 37°C for 1 h. Counterstaining of cells was achieved through 4′,6‐diamidino‐2‐phenylindole (MBD0020, Sigma‐Aldrich). Images for immunofluorescence were acquired through a fluorescence microscope (ECLIPSE E800), with five random fields obtained per sample. Quantification of DAPI‐positive nuclei and γH2AX‐positive cells was conducted via the use of ImageJ software. γH2AX‐positive cells were identified as cells containing more than 10 foci of γH2AX staining in their nuclei.^25^, ^26^

### 2.13 Bromodeoxyuridine pulse‐chase experiment

Cells underwent pulsing with 10 µM bromodeoxyuridine (BrdU) for 30 min, subsequent washing using media containing 10 µM thymidine, irradiation, and then collection at diverse time points. Processing and analysis of the cells involved treating them with the anti‐BrdU antibody (SAB4700630, Sigma‐Aldrich LLC) and FITC‐conjugated anti‐mouse antibody followed by propidium iodide staining. Sample analysis was performed using FlowJo software on FACSAria Fusion flow cytometer (BD Biosciences).^27^

### 2.14 Analysis of homologous recombination (HR) and non‐homologous end joining (NHEJ) Repair

As previously mentioned,^28^, ^29^ assessment of homologous recombination (HR) and non‐homologous end joining (NHEJ) was conducted in cells. Transient transfection of HEK293T cells with NHEJ‐GFP or HR‐GFP reporter plasmids, along with Vector, TTN‐MUT, pcDNA3.1 (oe‐NC), or pcDNA3.1‐ANKRD1 (oe‐ANKRD1) plasmids, was carried out for 24 h. Subsequently, an adenovirus containing I‐SceI was added to induce DSBs in the constant sequence of the reporter plasmid. To clarify, NHEJ‐GFP‐PEM1 or HR‐GFP‐PEM1 plasmids were transiently co‐transfected with pcDNA3.1 and mCherry plasmids applying Lipofectamine 2000 as a transfection control for GFP‐positive cells. The introduction of DSBs at the recognition sequence within the reporter construct was triggered by infecting cells with an adenovirus that carried I‐SceI restriction endonuclease, following a 24 h incubation period. The medium with the virus was exchanged for a regular cell culture medium after 18 h. Post 24 h period, the proportion of GFP‐HR and GFP‐NHEJ positive cells was analyzed using the LSRII flow cytometer.

### 2.15 Chromatin immunoprecipitation

Chromatin immunoprecipitation (ChIP) experiments were executed with the SimpleChIP Enzymatic Chromatin IP Kit (Magnetic Beads) (#9003, Cell Signaling Technology). Cells were grown to 95% confluency, and approximately 3 × 10^7^ cells underwent cross‐linking using 1% formaldehyde for 10 min at room temperature, and the cross‐linking reaction was halted by adding glycine solution. The cells were rinsed with cold PBS two times before being suspended in cold PBS along with a protease inhibitor cocktail. Fifteen rounds of sonication, each lasting 10 s, were employed on the cell lysates to produce DNA fragments of 200−300 bp in size. Subsequently, overnight incubation at 4°C with 5 µg of JUN antibody (ab40766, 1:20, Abcam) and 5 µg of normal IgG (ab172730, Abcam) was performed on the sheared chromatin, followed by a 2 h incubation with 30 µL of protein G magnetic beads. The immunoprecipitates underwent three rounds of washing with a low salt wash buffer, one round with a high salt wash buffer, and then underwent elution and reverse cross‐linking using 6 µL of 5 M NaCl and 2 µL of proteinase K at 65°C for 2 h. Finally, the utilization of purification columns facilitated the process of DNA purification. The extracted DNA was analyzed by qPCR using primers targeting ANKRD1.^30^

### 2.16 Bioluminescence reporter gene experiment

Two constructs were created: PGL3‐basic‐ANKRD1‐WT, containing the ANKRD1 bioluminescence reporter gene, and PGL3‐basic‐ANKRD1‐MUT, carrying a mutation at the JUN binding site. These reporter plasmids, along with the control plasmid oe‐JUN, were co‐transfected into HCT116^WT/−^, HCT116^−/MUT^, SW837^WT/WT^, and SW837^WT/MUT^ cells. After transfection for 24 h, centrifugation at 10 000 g for 1 min was performed after cell lysis, and then the supernatant was harvested. Measurement of luciferase activity for the ANKRD1 promoter was carried out with the dual‐luciferase reporter assay system (E1910, Promega). The Firefly luciferase activity in each cell sample was detected by incubating with 100 µL of Firefly luciferase working solution, while the Renilla luciferase activity was measured by incubating with 100 µL of Renilla luciferase working solution. RLU1 represents the Firefly luciferase reaction intensity, RLU2 corresponds to the internal control Renilla luciferase reaction intensity, and the proportion between the two data sets was computed as RLU1/RLU2.^31^

### 2.17 RIP‐qPCR

Preceding RIP‐qPCR, a single cold PBS wash was performed on the cells, followed by their collection in 2 mL cold PBS within a 10 cm culture dish. Nuclear extraction was carried out on these cells, followed by sonication. Subsequently, 2 µg of JUN antibody (ab40766, 1:20, Abcam) or the corresponding control rabbit IgG (ab172730, Abcam) was conjugated with protein A/G magnetic beads (88802, Thermo Fisher Scientific) and incubated at 4°C for 4 h. After washing thrice, the beads were incubated overnight at 4°C with precleared nuclear extracts in RIP buffer (150 mM KCl, 25 mM Tris pH 7.4, 5 mM EDTA,  .5 mM DTT,  .5% NP40, 1× protease inhibitor, 10 U/mL RNase inhibitor). After the incubation period, the beads underwent three washes with RIP buffer, were then resuspended in 80 µL of PBS, and underwent DNA digestion at 37°C for 30 min. This was followed by the addition of 2 µL of proteinase K (25530031, 20 mg/mL, Thermo Fisher) and incubation at 55°C for another 30 min. Lastly, the ANKRD1 promoter levels were assessed through quantitative PCR after extracting RNA from both Input and IP samples employing the TRIzol method (primers: forward: 5′‐CTTTTAAAGGTGGCCCTTCC‐3′, reverse: 5′‐CAGCTTTGAGATGCAAACCA‐3′).^32^

### 2.18 RT‐qPCR

Trizol reagent (15596026, Invitrogen) was employed for total RNA isolation, following the prescribed guidelines for extraction. The PrimeScript RT reagent Kit (RR047A, Takara) was utilized for reverse transcribing RNA into cDNA. The RT‐qPCR analysis was applied to the synthesized cDNA utilizing the Fast SYBR Green PCR kit (11736059, Thermo Fisher Scientific (China) Co., Ltd.), with three replicates allocated per sample. GAPDH served as the reference marker in the analysis. The calculation of relative expression levels was based on the 2^−ΔΔCt^ methodology. The experiment was duplicated thrice. Table S1 contains the primer sequences utilized for RT‐qPCR in this investigation (primers produced by Takara).

### 2.19 Co‐immunoprecipitation

Protease inhibitors (Sigma‐Aldrich) and PhosSTOP phosphatase inhibitors (Roche) were both supplemented in all buffers. Cells were collected in pre‐chilled Buffer A (10 mM HEPES (pH 7.8), 1.5 mM MgCl₂, 10 mM KCl, 20 mM ZnCl₂) and diluted in 10 volumes of pre‐chilled Buffer B (150 mM NaCl, 50 mM Tris (pH 7.5),  .1% SDS, 1% NP‐40) for lysis. The lysates underwent pre‐clearing with IgG Sepharose 6 Fast Flow (GE Healthcare) for 1 h and then immunoprecipitated with 4 mg of antibody, after which they were adsorbed onto protein G Sepharose 4 Fast Flow (GE Healthcare). Mouse‐anti‐JUN (610326, BD Biosciences) and anti‐IgG (sc‐51993, Santa Cruz Biotechnology) were used as controls. The precipitates were washed three times with pre‐chilled Buffer B and eluted with 250 mM Tris (pH 6.8), 10% glycerol, 4% SDS, 2% β‐mercaptoethanol, and  .006% bromophenol blue. Subsequent to SDS‐PAGE, the proteins were assessed via Western blot analysis.^33^

### 2.20 Detection of protein expression in cells using Western blot

Extraction of total protein from cells involved the utilization of RIPA lysis buffer (P0013C, Beyotime) with the incorporation of PMSF. The procedure for extraction involved placing the cells on ice for 30 min at 4°C, then centrifuging at 8000*g* for 10 min to procure the supernatant. Evaluation of total protein concentration was performed with a BCA assay kit (23227, ThermoFisher, USA). Subsequently, conducting SDS‐PAGE gel electrophoresis after dissolving 50 µg of protein in 2× SDS loading buffer and boiling at 100°C for 5 min. Transfer of the proteins to a PVDF membrane was followed by blocking at room temperature for 1 h with 5% non‐fat milk powder. The PVDF membrane underwent overnight incubation at 4°C with appropriately diluted primary antibodies against TTN (1:2000, ab284860, Abcam), cleaved PARP (1:2000, ab32064, Abcam), cleaved caspase 3 (1:1000, ab2302, Abcam), γH2AX (1:2000, ab26350, Abcam), RAD51 (1:1000, ab133534, Abcam), PARP (MA5‐15031, 1:1000, ThermoFisher), ANKRD1 (1:500, ab64963, Abcam), and β‐actin (ab8226, 1:2500, Abcam). The membrane underwent three successive 10 min washes with TBST post‐incubation and was subsequently exposed to HRP‐conjugated secondary antibodies, goat anti‐rabbit or anti‐mouse IgG H&L (HRP) (ab6721/ab6728, 1:2000, Abcam), for 1 h at room temperature. Upon completion of TBST washes, the membrane was situated on a clear glass plate. ECL chemiluminescent reagents (abs920, Elabscience Biotechnology Co. Ltd.) components A and B were combined in darkness, applied onto the membrane, and visualized with the Bio‐Rad imaging system (Bio‐Rad). Quantification of the band density in the Western Blot images was conducted using the Image J analysis software, with β‐actin acting as the loading control protein. The experiments underwent three independent repetitions in total.^34^

### 2.21 In vivo experiments

Acquired from Beijing Vital River Laboratory Animal Technology Co., Ltd. were female BALB/c nude mice, BALB/c mice, and C57BL/6 mice (6–8 weeks old). Adhering to the current regulations and standards governing the use of experimental animals in China, the Beijing Hospital Animal Care Committee approved all animal experiments (approval no. BJH‐AE‐201901008).

Female BALB/c nude mice, BALB/c mice, and C57BL/6 mice were used to establish subcutaneous tumour models by injecting modified human READ HCT116 and SW837 cells into BALB/c nude mice, mouse cell line CT26 into BALB/c mice, and MC38 into C57BL/6 mice subcutaneously on the right side, which led to the formation of subcutaneous tumours.^26^ Once the xenografts reached a volume of approximately 100 mm^3^, the mice underwent X‐ray treatment of 2 Gy/day for seven consecutive days (total of 14 Gy). Lead sheets were used to shield the body of the mice except in the area with xenografts. The advancement of tumour growth was tracked, and the volume of the tumour was computed according to the stipulated formula: tumour volume V = (LW^2^)/2. Upon completing 5 weeks of radiotherapy, mice were euthanized using CO_2_, and tumours were dissected, measured, photographed, weighed, and subjected to further analysis.

Furthermore, male C57BL/6 mice received subcutaneous injections of WT and TTN‐KO MC38 tumour cells (3 × 10^5^) into the bilateral flanks for T‐cell depletion experiments. To deplete T cells, antibodies were injected before tumour inoculation, following the manufacturer's instructions, with 200 mg of Anti‐CD8 (BE0061, Bio X Cell), 200 mg of Anti‐CD4 (BE0003‐1, Bio X Cell) neutralizing antibody, or 200 mg of isotype IgG2b antibody (BE0090, Bio X Cell) via tail vein injection at noon on days 2, 4, 6, 9, and 12. Once the xenografts reached a volume of approximately 100 mm^3^, the mice received 12 Gy X‐ray treatment.^35^, ^36^, ^37^

### 2.22 Analysis of immune cells in the intra‐tumoral microenvironment

After subcutaneous injection of WT and TTN‐KO CT26 or MC38 tumour cells into BALB/c and C57BL/6 mice, flow cytometry analysis was performed on the post‐mortem tumour tissues. The tissues underwent an overnight staining procedure with Alexa Fluor® 647 Anti‐CD4 (ab225310, 1:250, Abcam) and Alexa Fluor 647 Anti‐CD8 (ab237365, 1:100, Abcam) antibodies. The BD LSR Fortessa cell analyzer (BD Biosciences) was employed for collecting fluorescence data, followed by analysis employing FlowJo version 10. The calculation involved dividing the number of positive cells analyzed in the flow cytometry by the total cell count, and the quantitative assessment focused on the percentage of positive cells.

### 2.23 Immunohistochemistry (IHC) staining

Immunohistochemistry (IHC) involves the analysis of tumour tissue at the endpoint. Following fixation with 4% paraformaldehyde for 24 h, paraffin‐embedded tissues were then sectioned into slices measuring 3 µm. After baking at 60°C for 1 h, the slices underwent deparaffinization in xylene I and xylene II, dehydration in graded alcohol, treatment with 3% hydrogen peroxide at room temperature for 20 min to halt endogenous peroxidase activity, washing with PBS, and blocking with 10% goat serum for 15 min. Primary antibodies against Ki67 (ab231172, Abcam), γH2AX (ab124781, Abcam), CD4 (ab288724, Abcam), and CD8 (ab316778, Abcam) were added and incubated overnight at 4°C, after which PBS was used for three rounds of washing. The following step involved incubating the sections with the secondary antibody, biotinylated goat anti‐rabbit IgG (ab97051, 1:2000, Abcam), at room temperature for 40 min. After washing with PBS, visualization was achieved using DAB (DA1010, Solarbio, China) for 10 min. Counterstaining with hematoxylin (H8070, Solarbio) lasted 1 min, followed by rinsing with running water, alcohol dehydration, xylene clearing, and coverslipping with neutral resin. The negative control involved the use of PBS in place of the primary antibody. The final results were assessed independently by two blinded observers, and five regions of high power were randomly picked through an optical microscope. Image J software was utilized to analyze the percentage of positive cells. Sample staining was automatically scored by applying the IHC Profiler plugin, followed by counting positive and negative cells using the Trainable Weka Segmentation plugin.^38^

### 2.24 TUNEL staining

Quantification and identification of apoptosis in tissue cells were achieved through the conduct of the TUNEL assay using the Cell Apoptosis Detection Kit (C1088, Beyotime). Initially, tissue sections were deparaffinized and rehydrated, followed by a 30 min pretreatment with Proteinase‐K (ST532, Beyotime) without DNase to sensitize the tissue sections. Subsequently, incubation of the tissue sections with the TUNEL detection solution from the kit was carried out at 37°C for 1 h. A fluorescent microscope was used to examine the slides after sealing with an anti‐fade mounting medium.^39^

### 2.25 Statistical analysis

Utilization of the statistical software SPSS 24.0 (SPSS, Inc.) facilitated data processing. Normality and homogeneity of variance tests were executed across all data points. Mean ± standard deviation were utilized to present data for continuous variables with a normal distribution. Utilizing independent sample *t*‐tests, comparisons were made between two groups, with comparisons among multiple groups conducted using one‐way analysis of variance followed by Tukey's post‐hoc test. The technique of repeated measures analysis was utilized to assess data across various time intervals during experiments. Statistically significant distinctions were denoted by a *p*‐value below  .05.

## 3 RESULTS

### 3.1 TTN mutation may be a potential immunogenic mutation in READ

**FIGURE 1:**
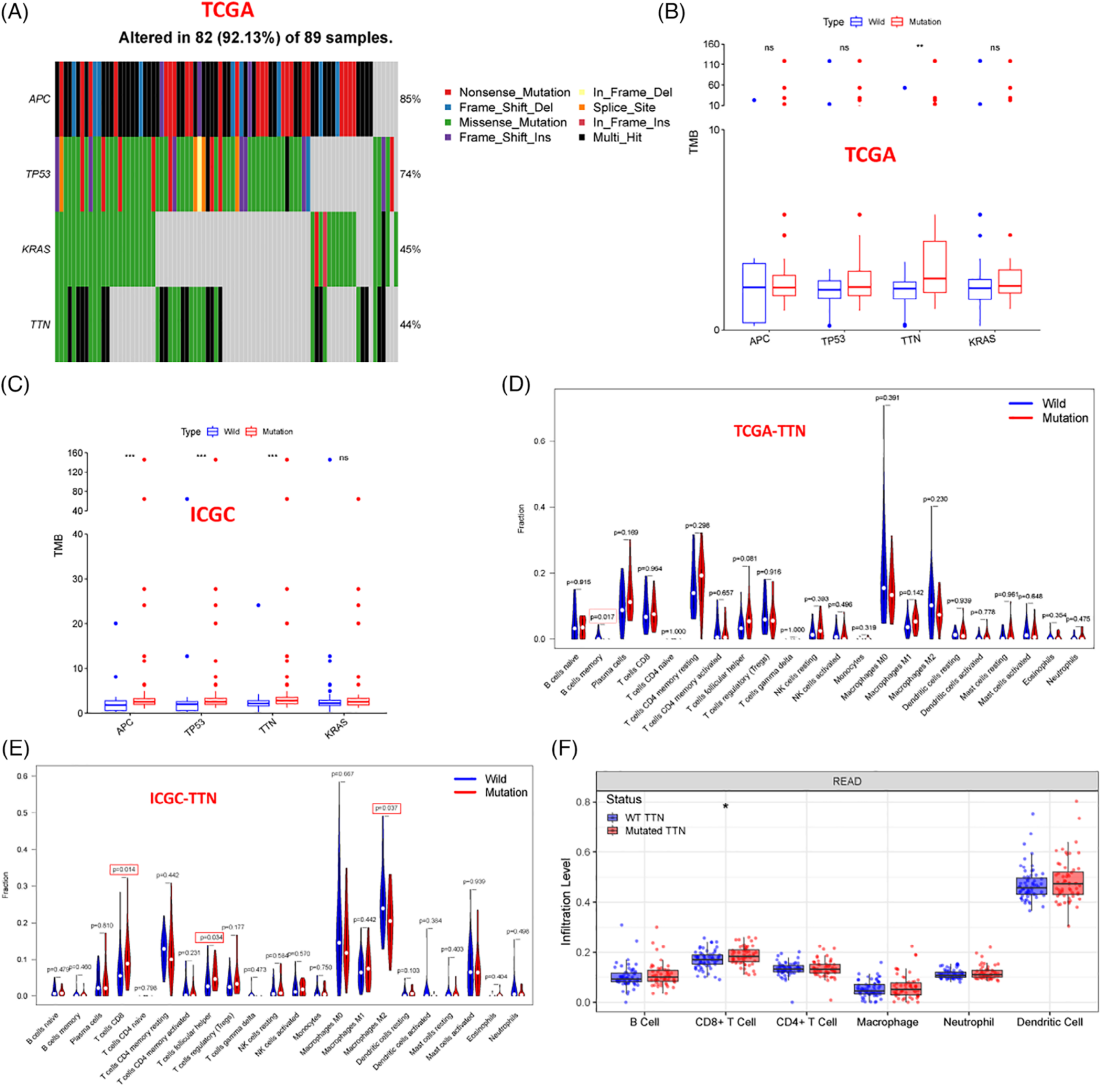
Correlation Analysis between TTN Mutation in READ and TMB as well as immune cell infiltration. (A) Mutation frequencies of APC, TP53, KRAS, and TTN in TCGA‐READ samples (*N* = 89) are presented. (B, C) Differential expression analysis of APC, TP53, KRAS, and TTN mutations in TCGA‐READ (*N* = 89) or ICGC‐READ (*N* = 134) samples with respect to TMB. (D, E) Relationship between TTN mutation in TCGA‐READ (*N* = 89) or ICGC‐READ (*N* = 134) samples and tumour‐infiltrating immune cells in the READ microenvironment. (F) Analysis of TTN gene mutation levels in the TIMER database (https://cistrome.shinyapps.io/timer/) and their differential effects on six types of immune cells. ‘ns’ indicates no significant difference, *p < .05, **p < .01, and ***p < .001.

To explore the genetic mutation status of READ, we initially downloaded the somatic mutation spectrum of 89 READ patients from TCGA. The visualization of READ mutation data was conducted using the “maftools” package. As depicted in Figure S1A, the mutation information of each gene in each sample was presented in a waterfall plot, revealing that among the 89 READ samples, 85 cases (85/89, 95.51%) exhibited somatic mutations. The genes with the highest mutation frequencies in these samples were APC, TP53, KRAS, TTN, and MUC16, with mutation frequencies of 85%, 74%, 45%, 44%, and 27%, respectively. In Figure S1B, it was observed that missense mutations were predominantly found in READ samples, with a higher prevalence of SNPs compared with insertions or deletions, and specifically, the most prevalent SNV observed was C > T. Furthermore, an analysis of genetic mutations from 134 patients in the United States was performed using data from the ICGC database. Figure S1C displays the top 30 genes in the mutation library, where APC, TP53, KRAS, and TTN exhibited the highest mutation frequencies. Among the two datasets, a comparison of the top 20 genes revealed that 15 genes were shared (Figure S1D), including APC, TP53, KRAS, and TTN, which exhibited relatively high mutation frequencies in both TCGA and ICGC datasets. Detailed mutation frequencies in TCGA are presented in Figure 1A. Subsequently, our analysis focused on these mutated genes in the follow‐up investigations.

Furthermore, we calculated the TMB as the number of mutation events per million bases in tumour samples. Based on a pan‐cancer analysis of TCGA data, READ samples ranked high in TMB (Figure S2A). The relationship between the mutations of APC, TP53, KRAS, TTN and TMB in READ samples from TCGA and ICGC was further analyzed, revealing that only TTN mutation was significantly associated with elevated TMB in TCGA and ICGC‐READ samples (Figure 1B,C).

Subsequently, the investigation into the link between TTN mutation and tumour‐infiltrating immune cells in the READ microenvironment was conducted through the utilization of the CIBERSORT algorithm. Calculating the ratios of 22 immune cell infiltrates within the tumour microenvironment is the main function of this algorithm. A comparison of immune cell expression differences between TTN‐WT and TTN‐MUT groups showed higher infiltration scores of memory B cells in the TTN‐MUT group when contrasted with the TTN‐WT group in TCGA‐READ samples, while in ICGC‐READ samples, the TTN‐MUT group exhibited higher infiltration scores of CD8^+^ T cells and follicular helper T cells and lower infiltration scores of M2 macrophages compared with the WT group (Figure 1D,E). Additionally, correlation analysis based on the TIMER database revealed a substantial escalation in CD8^+^ T cell immune cell infiltration in the TTN‐MUT group (Figure 1F). Moreover, positive correlations were observed between the levels of TTN expression and the presence of CD4^+^ T cells, CD8^+^ T cells, B cells, macrophages, neutrophils, and DCs in the tissue (Figure S2B). Furthermore, survival analysis based on clinical data from TCGA‐READ indicated that although no substantial variance was observed in overall survival between the TTN‐WT and TTN‐MUT groups, there was a trend toward benefit (Figure S2C).

Overall, these results suggest that TTN mutation in READ is significantly associated with TMB and CD8^+^ T‐cell infiltration, indicating a potential immunogenic antigenic mutation.

### 3.2 TTN gene mutations enhance radiosensitivity in READ cells

Based on pathway enrichment analysis of mutation data from TCGA‐READ samples, we found that these mutated genes are enriched in cell cycle‐related pathways, which are associated with the growth of cancer cells (Figure S3A). DSBs represent the most severe type of DNA damage induced by radiotherapy in cancer cells, often leading to cell apoptosis, autophagy, and cell cycle arrest.^40^ Thus, to examine the possible effects of TTN mutations on radiotherapy, we utilized the CRISPR/Cas9 editing system to construct TTN‐R5163H heterozygous and homozygous knock‐in mutants in HCT116 and SW837 cells, respectively. To confirm the presence of TTN mutations in these cell lines, Sanger sequencing of TTN exon 1 was conducted on genomic DNA isolated from HCT116^TTN WT/−^ (‘HCT116^WT/−’^), HCT116^TTN−/MUT^ (‘HCT116^−/MUT’^), SW837^TTN WT/WT^ (‘SW837^WT/WT’^), and SW837^TTN WT/MUT^ (‘SW837^WT/MUT’^) cells. In HCT116, HCT116^−/MUT^ cells showed a single transition from G to A in the codon. In SW837, detected in the codon of SW837 WT/MUT cells were both G and A, suggesting a heterozygous mutation (Figure S3B).

**FIGURE 2:**
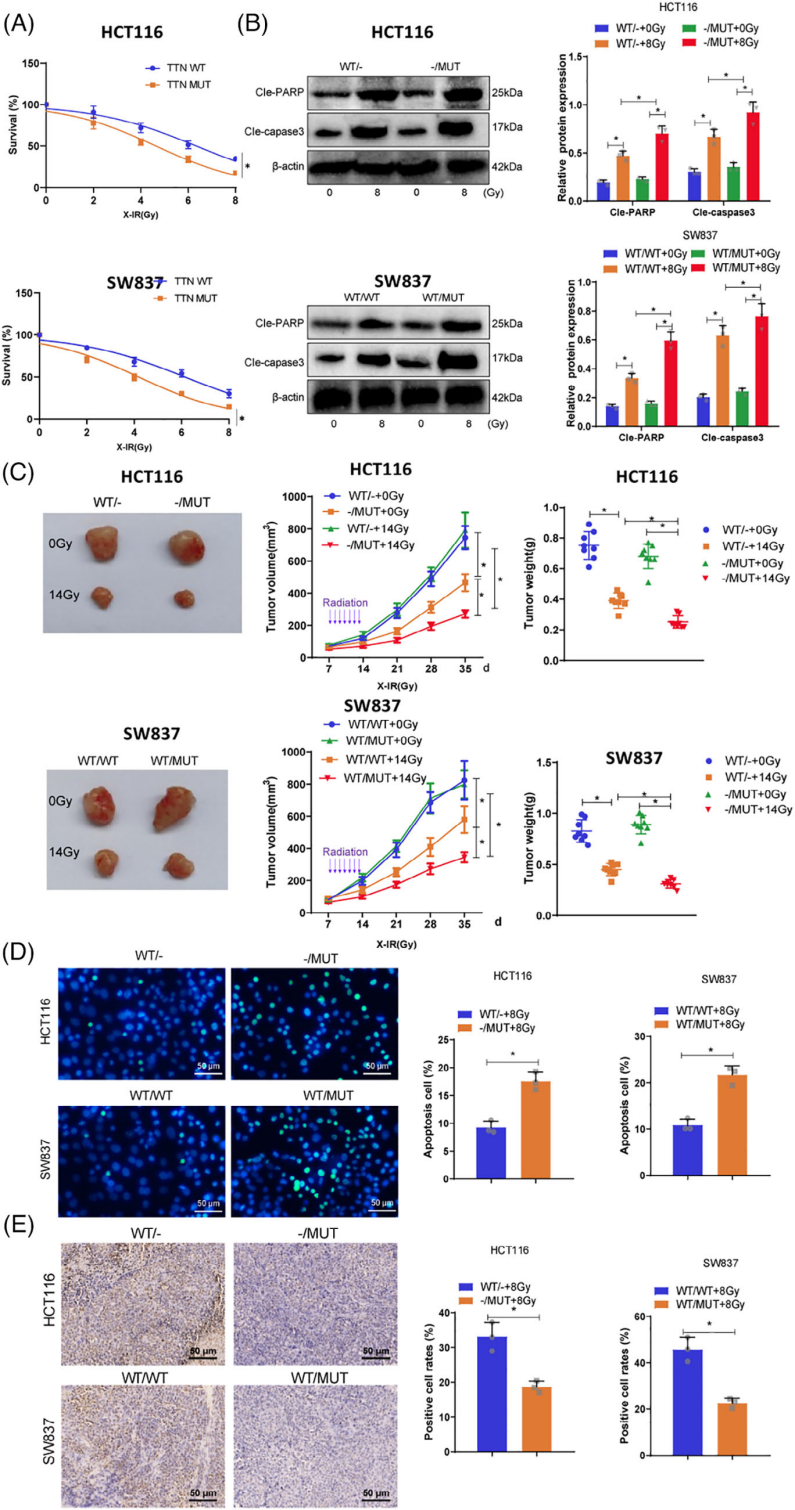
The impact of TTN mutations on radiosensitivity in vitro and in vivo. (A) Cell survival rate detected by radiation clonogenic survival assay. (B) Protein expression of cleaved‐PARP and cleaved‐caspase 3 in cells determined by Western blot. (C) Comparison of tumour volume and weight in BALB/c nude mice. (D) Cell apoptosis detected by TUNEL fluorescence assay (scale bar = 50 µm). (E) Expression of Ki67 examined by IHC (scale bar = 50 µm). *p < .05. Cell experiments were repeated three times, and ten mice were used in the tumour formation experiments.

To assess whether TTN mutations affect the functionality of READ cells, we examined the cancer cell functions of TTN wild‐type (HCT116 WT/− and SW837 WT/WT) and TTN mutant type (HCT116−/MUT and SW837 WT/MUT cells) within 24 h. The results showed no significant changes in cell proliferation, invasion, and migration abilities between TTN wild‐type and mutant cells (Figure S4). Subsequently, to determine the impact of TTN mutations on cell radiosensitivity, TTN wild‐type and mutant allele cells were exposed to escalating doses of X‐irradiation and underwent a standard radiation clonogenic assay. The results revealed that compared with TTN WT cells, TTN MUT cells exhibited lower survival rates post‐irradiation (Figure 2A). Furthermore, elevated levels of cleaved‐PARP and cleaved‐caspase 3 were confirmed through Western blot analysis in response to TTN mutations (Figure 2B).

Next, the effect of TTN mutations on radiosensitivity in vivo was examined using a subcutaneous xenograft model in BALB/c nude mice. When tumours derived from HCT116−/MUT and HCT116 WT/− cells reached 100 mm^3^, treatment options for the tumours included daily radiation therapy or no intervention. Figure 2C demonstrated that, after radiation treatment, both HCT116 WT/− and HCT116−/MUT tumours exhibited varying degrees of growth inhibition, with notably more significant tumour suppression in HCT116−/MUT+14 Gy compared with HCT116 WT/‐+14 Gy. Similarly, in SW837 cells, tumours from the WT/MUT group showed significant reductions in size and weight compared with the WT/WT group. TUNEL staining and Ki67 staining were used to assess apoptosis and proliferation rates in tumour cells. The results indicated increased radiation‐induced apoptosis in the TTN‐MUT group (Figure 2D), along with significantly lower Ki67‐positive cells in the mutant group, suggesting escalated radiation‐related injuries in the mutant group (Figure 2E). These findings collectively demonstrate the radiosensitivity of TTN mutations both in vitro and in vivo.

### 3.3 TTN mutation impairs DNA damage repair in radiation response

**FIGURE 3:**
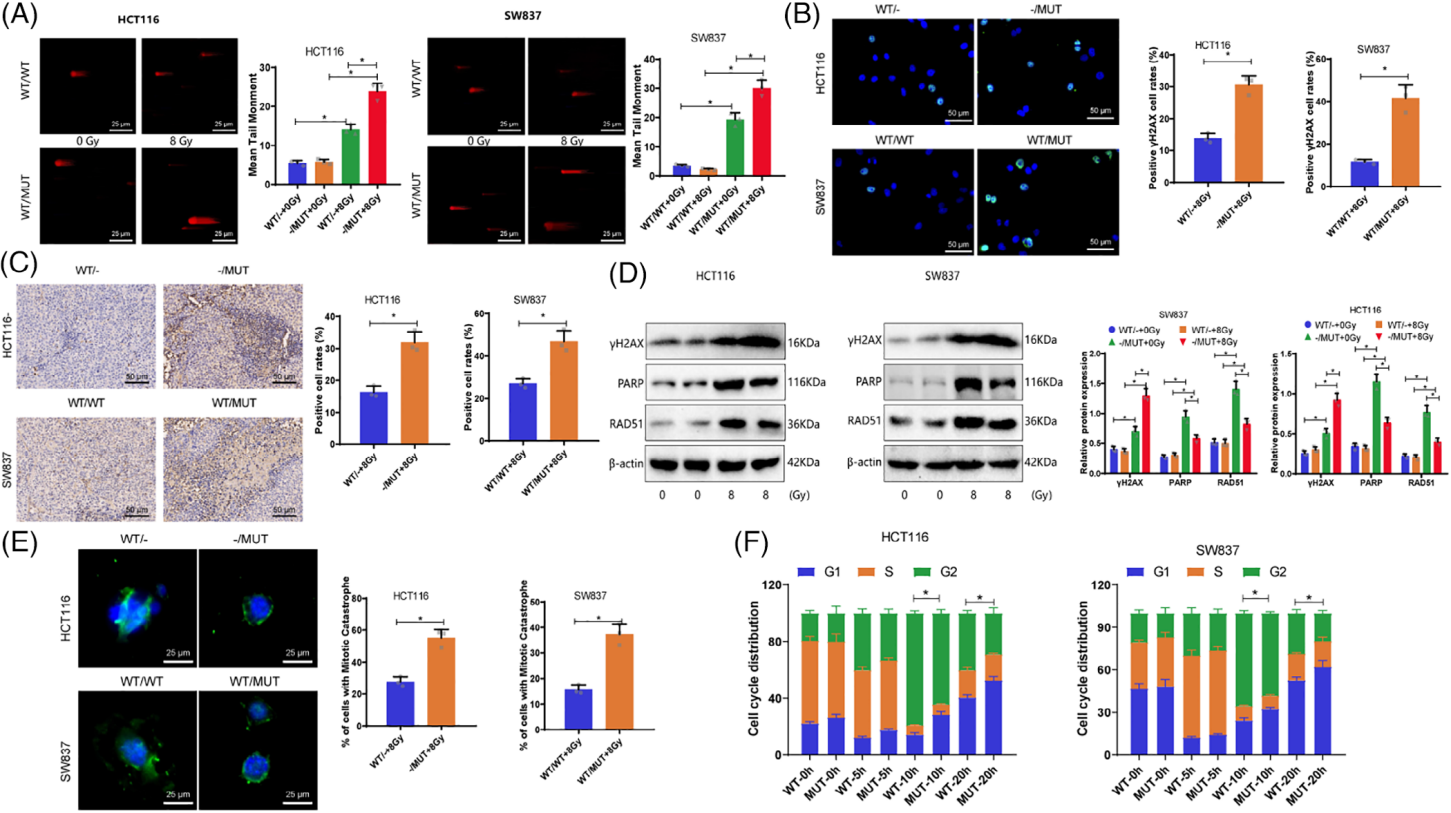
TTN mutation impact on DDR in radiation reaction. (A) Neutral comet assay to detect cellular DNA damage extent; (B) Immunofluorescence assay to detect γH2AX expression, with blue DAPI fluorescence marking cell nuclei and green indicating γH2AX (scale bar = 50 µm); (C) IHC to detect γH2AX expression in tumour tissues from subcutaneously transplanted BALB/c nude mice (scale bar = 50 µm). (D) Western blot assay to detect protein expression of γH2AX, PARP, and RAD51. (E) Immunofluorescence assay to detect mitotic mutations (scale bar = 25 µm). (F) Analysis of cell cycle distribution using BrdU incorporation method. *p < .05. Cell experiments were repeated thrice, and mouse tumourigenesis experiments had *n* = 10.

The main process leading to radiation‐induced cell death involves the creation of DSBs followed by mitotic cell death.^41^ The effective and trustworthy repair of DNA damage is vital for the survival of normal cells but can also serve as a mechanism for treatment resistance in tumour cells. Assessment of how TTN mutation influences the overall DNA repair process in cells following irradiation was carried out through the performance of neutral comet assays, revealing a significant increase in tail moment in HCT116 ‐/MUT and SW837 WT/MUT cells at 24 hours, indicating a change in DNA damage response (DDR) in TTN MUT cancer cells (Figure 3A).

The dynamics of DNA damage generation and repair of DSB can be indicated by γH2AX.^42^, ^43^ Immunofluorescence results showed that 24 h after irradiation, the fluorescence intensity of γH2AX in TTN MUT cells was significantly higher than in TTN WT cells (Figure 3B). Immunohistochemical analysis of xenograft tumour tissues in nude mice demonstrated an increased expression of γH2AX in the TTN mutation group (Figure 3C). Further evaluation of protein levels involved in DNA damage and repair processes through Western Blot revealed a significant increase in γH2AX protein expression in TTN MUT cells at 24 h post‐irradiation, while DNA damage repair‐related proteins RAD51 and PARP exhibited decreased expression, suggesting that TTN mutation impedes the DNA damage repair process induced by radiation (Figure 3D).

The impact of IR leads to mitotic catastrophe as a prevailing form of cell demise. To further assess the impact of TTN mutation on cellular DNA damage, the rate of mitotic catastrophe occurrence was determined at 72 h post‐IR. We observed a higher level of mitotic catastrophe associated with the presence of TTN mutation in HCT116 and SW837 cells (Figure 3E). Additionally, the sensitivity to IR is largely determined by the regulation of the cell cycle, with cells being most sensitive during the G2/M phase and showing decreased sensitivity in the G1 phase.^44^ Cells are significantly arrested in the G2/M phase to repair IR‐induced DNA damage. We initially compared the cell cycle distribution of asynchronously growing cells with two alleles. TTN mutation cells exhibited a slightly lower proportion of G1 phase cells and a significantly higher proportion of G2/M cells when juxtaposed with wild‐type TTN cells (Figure S5). By further tracking the cell cycle advancement of IR‐labeled S‐phase HCT116 and SW837 tumour cells using BrdU labelling, we observed an increased G2/M phase arrest in TTN mutant cells at 10 and 20 h postirradiation (Figure 3F). These findings collectively suggest that TTN mutation can weaken DNA damage repair in the radiation response.

### 3.4 TTN mutation reduces ANKRD1 expression by disrupting JUN recruitment

To investigate the regulatory mechanism by which TTN mutations promote radiotherapy sensitivity, we utilized the STRING database to identify proteins interacting with TTN, setting the minimum interaction score at  .9. We identified at least 10 interacting proteins, namely NEB, TRIM63, TCAP, ACTN2, CAPN3, NBR1, FHL2, CMYA5, OBSCN, and ANKRD1 (Figure S6A). Among these, ANKRD1, a member of the ankyrin repeat protein family, has been documented to be pivotal in enhancing radiotherapy sensitivity in ovarian cancer, as evidenced by previous studies.^45^, ^46^, ^47^

**FIGURE 4:**
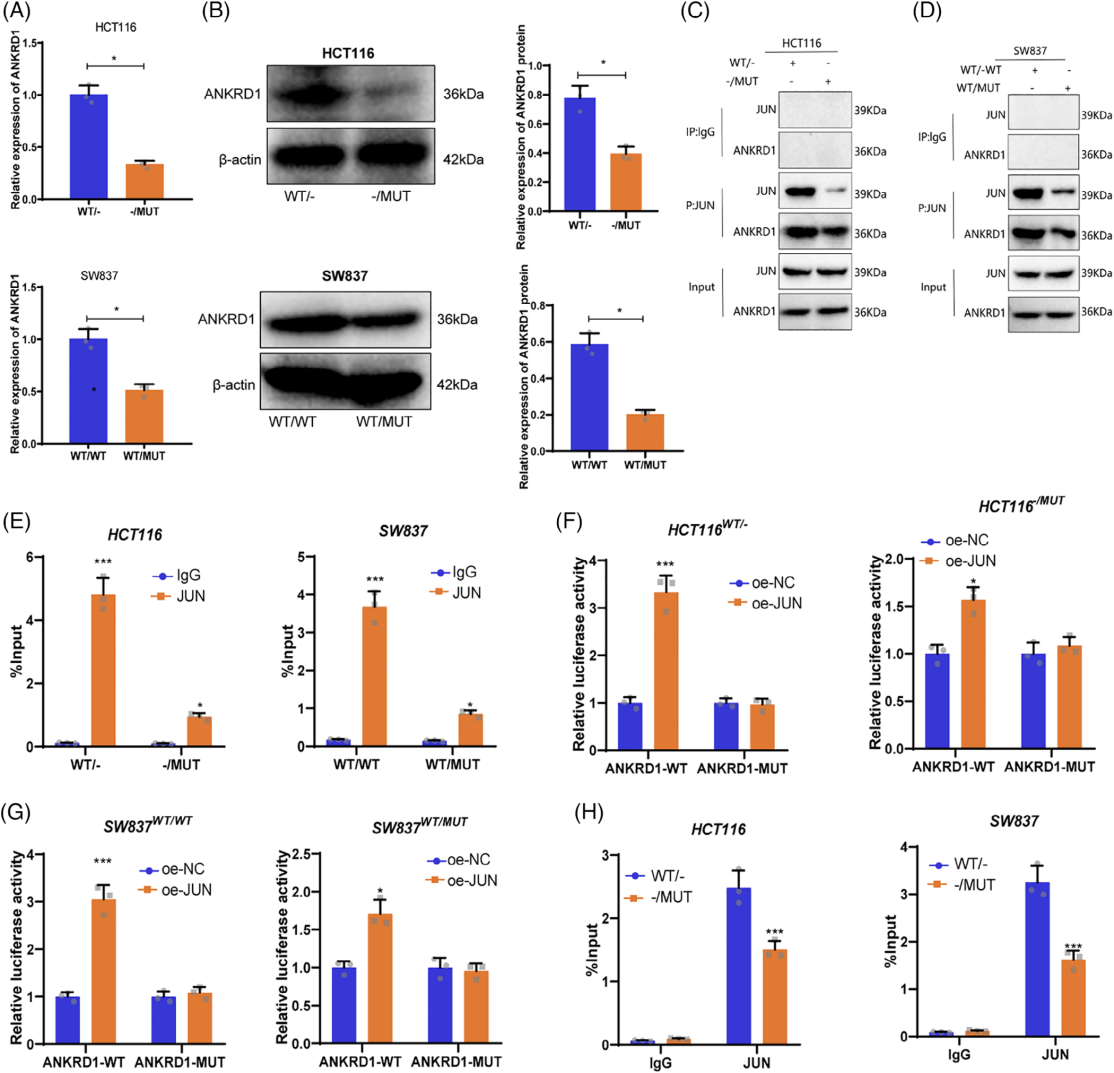
Impact of TTN mutation on ANKRD1 expression by modulating JUN recruitment. (A) ANKRD1 expression in SW837 and HCT116 allelic gene cells detected by RT‐qPCR. (B) Protein expression of ANKRD1 in cells detected by Western blot. (C, D) Co‐immunoprecipitation experiments examining the interaction between ANKRD1, JUN, and wild‐type or mutant TTN. (E) RIP‐PCR determines the binding of JUN to ANKRD1 mRNA in wild‐type and mutant TTN. (F, G) Dual‐luciferase reporter gene assay evaluating JUN's regulatory effect on ANKRD1 promoter activity. (H) ChIP‐qPCR detecting the enrichment of JUN in the ANKRD1 promoter region in wild‐type and mutant TTN; *p < .05, ***p < .001. Cell experiments were conducted in triplicate.

Further exploring the role of ANKRD1 in TTN mutations, analysis by qRT‐PCR validated decreased mRNA levels of ANKRD1 in HCT116 ‐/MUT and SW837 WT/MUT cells contrasted against wild‐type TTN cells (Figure 4A). At the protein level, ANKRD1 expression was significantly downregulated in TTN MUT allele tumour cells as well (Figure 4B).

The experimental results indicate that TTN mutations lead to a significant reduction in ANKRD1 expression. Given TTN's role as a large sarcomeric protein, it likely indirectly influences gene expression regulation. Therefore, we hypothesize that TTN may affect ANKRD1 expression by regulating transcription factors. JUN, a major component of the AP‐1 (Activator Protein 1) complex, is recognized for its vital involvement in the transcriptional regulation of many genes. ChIP‐seq data revealed the potential binding of JUN to the ANKRD1 promoter region, suggesting a direct regulation of ANKRD1 transcription by JUN (Figure S6B).

A series of experiments were designed to validate whether TTN modulates ANKRD1 expression through JUN recruitment. Co‐immunoprecipitation assays verified a physical interaction between ANKRD1 and JUN, showing a stronger interaction in wild‐type TTN cells compared with mutant TTN cells (Figure 4C,D). RNA immunoprecipitation experiments further confirmed the binding of JUN to ANKRD1 mRNA, with significantly higher binding in WT‐TTN cells than in MUT‐TTN cells (Figure 4E). Subsequent reporter gene assays assessed the regulatory effect of JUN on ANKRD1 promoter activity, revealing a significant upregulation of ANKRD1 promoter activity upon JUN overexpression, particularly pronounced in WT‐TTN cells (Figure 4F,G). ChIP‐qPCR results supported these findings, showing significantly higher enrichment of JUN at the ANKRD1 promoter region in WT‐TTN cells compared with MUT‐TTN cells (Figure 4H).

In summary, our study demonstrates that TTN mutation disrupts JUN recruitment, leading to decreased ANKRD1 expression.

### 3.5 ANKRD1 reverses TTN mutant cells’ sensitivity to radiation therapy

**FIGURE 5:**
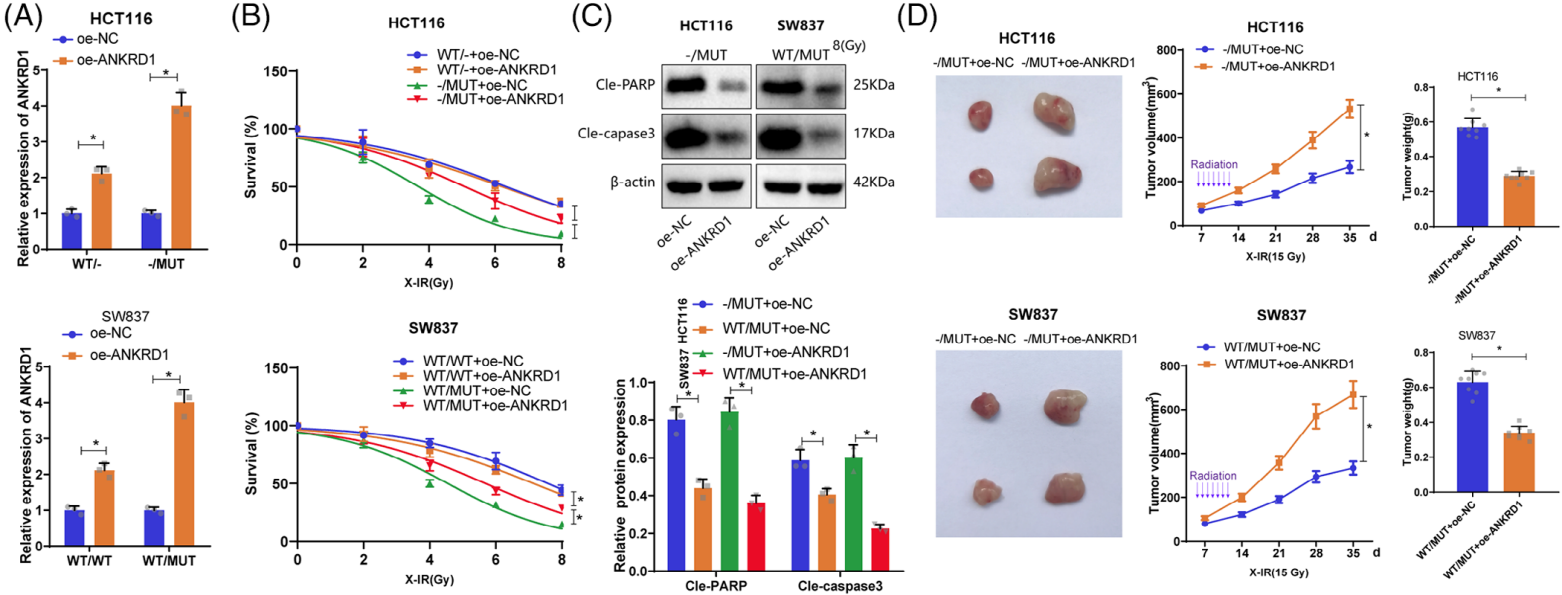
Deletion of ANKRD1 increases the radiosensitivity of TTN‐mutated cells. (A) RT‐qPCR was used to detect the expression of ANKRD1 in SW837 and HCT116 cells overexpressing ANKRD1. (B) Clonogenic assay assessed the impact of ANKRD1 overexpression on radiation sensitivity. (C) Western blot analysis was performed to measure the protein expression of cleaved‐PARP and cleaved‐caspase 3 in cells. (D) Tumour volume changes were evaluated in BALB/c nude mice xenografts. * indicates statistical significance at *p* < .05. Cell experiments were repeated three times, while the nude mice tumour experiments had *n* = 10 samples.

The research primarily focused on examining how ANKRD1 influences the radiosensitivity of TTN mutant tumour cells. Overexpression of the ANKRD1 gene led to a significant upregulation of ANKRD1 in both TTN wild‐type and mutant cells (Figure 5A), with protein levels shown in Figure S6C,D. Subsequent clonogenic assays revealed that ANKRD1 overexpression did not result in changes to the radiosensitivity of TTN‐WT cells, but notably reversed the sensitivity in TTN‐MUT cells (Figure 5B). In TTN‐MUT cells, post‐radiation treatment, the inhibition of cleaved‐PARP and cleaved‐caspase 3 expression was discovered by ANKRD1 (Figure 5C). In a BALB/c subcutaneous tumour model, it was observed that overexpression of ANKRD1 resulted in an increase in the volume and weight of TTN‐MUT tumours in contrast with the control group (Figure 5D). It was validated by Western blot analysis that ANKRD1 exhibited stable upregulation in TTN‐MUT tumours. These findings suggest that TTN mutation may modulate the radiosensitivity of READ cells through the downregulation of ANKRD1.

### 3.6 Reversal of reduced DNA damage repair capacity in radiation response by ANKRD1 in the presence of TTN mutations

**FIGURE 6:**
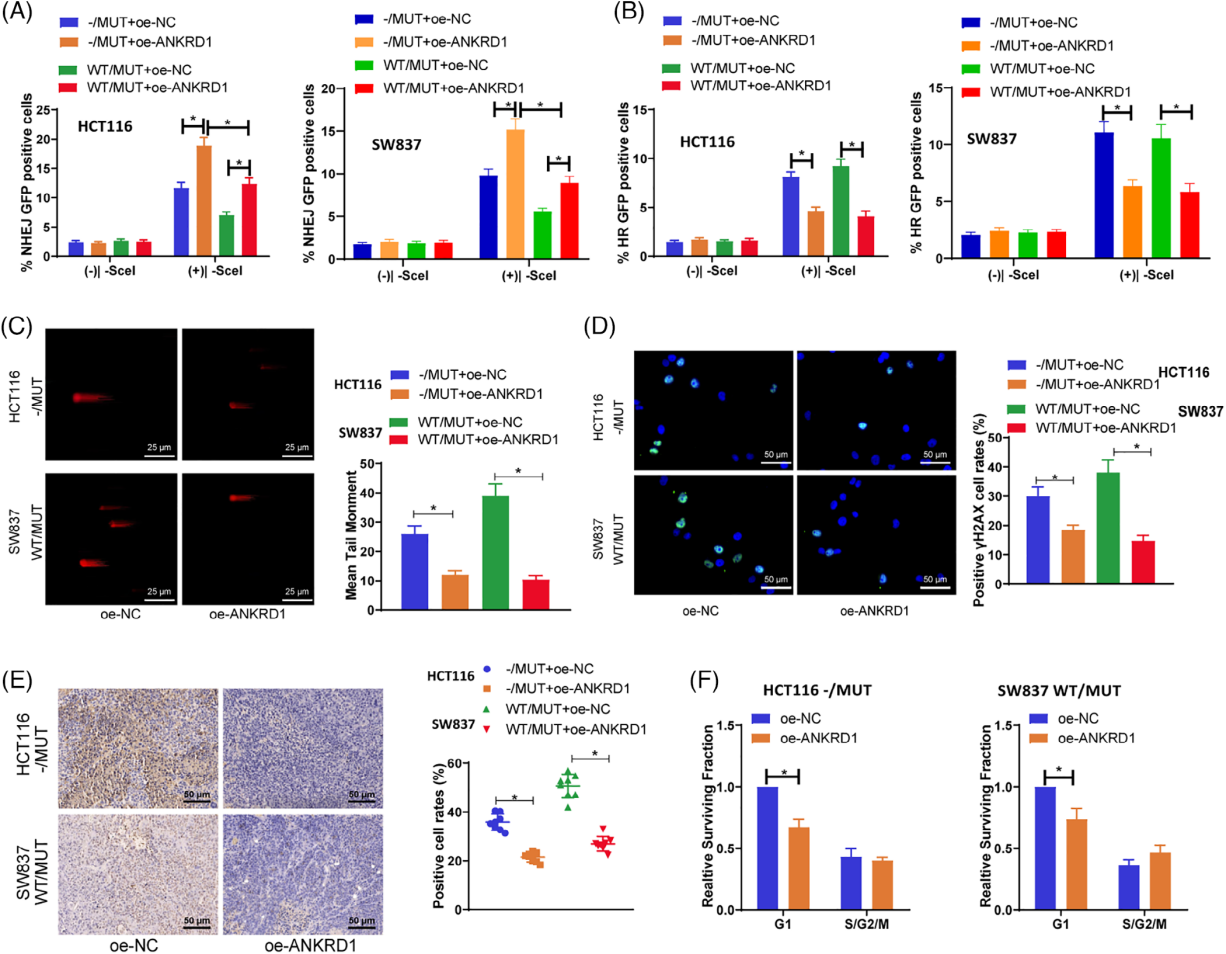
The influence of ANKRD1 on TTN mutation on DDR and repair capacity in radiation response. (A, B) The repair capacity of NHEJ or HR was detected by flow cytometry to measure GFP‐positive cells. (C) The extent of cellular DNA damage was assessed by neutral comet assay. (D) Immunofluorescence assay was conducted to detect γH2AX expression, with blue DAPI fluorescence labelling cell nuclei and green marking γH2AX (scale bar = 25 µm). (E) Immunohistochemical analysis detected γH2AX expression in tumour tissues of subcutaneously transplanted BALB/c nude mice (scale bar = 50 µm). (F) Cell cycle enrichment clone assay was performed to analyze cell cycle distribution. *p < .05. Cell experiments were repeated three times, and mouse tumour formation experiments had a sample size of *n *= 10.

We investigated whether ANKRD1 influences the impact of TTN mutations on the DDR in radiation. To more directly determine if TTN mutations alter cell survival by reducing ANKRD1 expression to modulate HR and/or NHEJ repair, we conducted transfections with both shared and separate expressions of ANKRD1. Plasmids expressing TTN‐MUT and repair reporter plasmids for NHEJ and HR (DR‐GFP/I‐SceI repair reporter) were used to assess HR and/or NHEJ repair capabilities. The results demonstrate that TTN MUT significantly reduces NHEJ while minimally affecting HR, and overexpression of ANKRD1 markedly increases the downregulation of NHEJ activity by TTN MUT (Figure 6A,B).

Comet assay results demonstrated a notable drop in tail moments in cells overexpressing ANKRD1 in relation to the oe‐NC group in TTN‐MUT cells (Figure 6C). Immunofluorescence analysis of γH2AX also indicated a reduction in γH2AX fluorescence in TTN MUT cells relative to the oe‐NC group upon overexpression of ANKRD1 (Figure 6D). Furthermore, similar outcomes were observed in an in vivo BALB/c nude mouse subcutaneous tumour transplantation model (Figure 6E). Cell cycle enrichment assays showed a significant increase in the G1 phase with ANKRD1 overexpression in TTN mutant cells (Figure 6F).

Overall, these findings suggest that ANKRD1 can reverse the diminished DDR and repair capabilities caused by TTN mutations in the context of radiation.

### 3.7 TTN deletion‐induced CD8^+^ and CD4^+^ T cell infiltration is essential for anti‐tumour immunity

**FIGURE 7:**
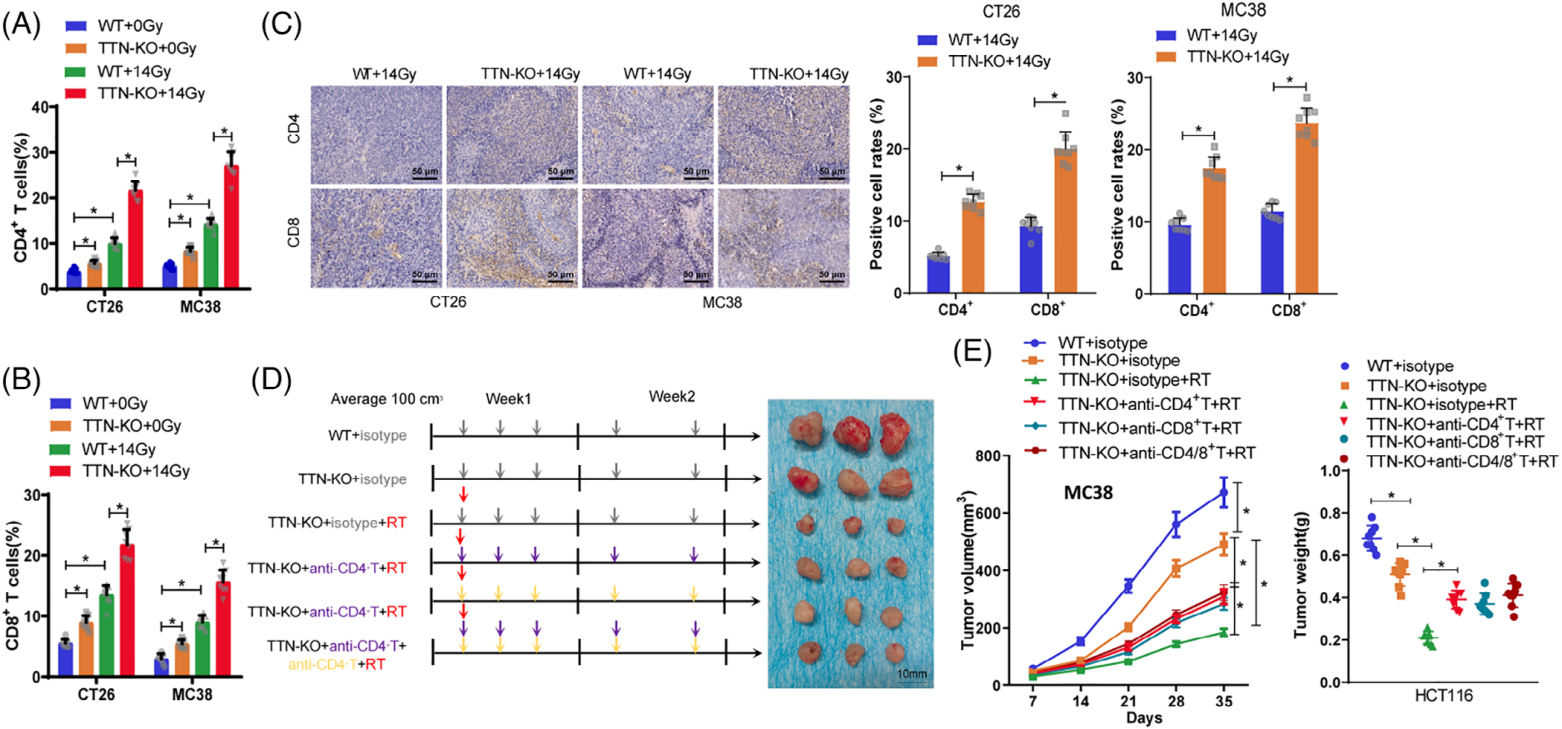
The impact of TTN knockout on immunostimulation in post‐radiotherapy subcutaneous xenograft mice models. (A, B) Flow cytometry analysis of the proportions of CD4^+^ and CD8^+^ T lymphocytes in tumour tissues of CT26‐inoculated BALB/c mice and MC38‐inoculated C57BL/6 mice, respectively. (C) Immunohistochemical analysis of the proportions of CD4^+^ and CD8^+^ T lymphocytes in tumour tissues of BALB/c and C57BL/6 mice (scale bar = 50 µm). (D) Schematic representation of neutralization assay. (E) Changes in tumour volume in C57BL/6 mice in different treatment groups. C57BL/6 mice inoculated with MC38 received one of the following treatments within a specified number of days: isotype control, isotype combined with radiotherapy and TTN knockout, anti‐CD8 neutralizing antibody therapy, anti‐CD4 neutralizing antibody therapy, and combined anti‐CD8 and anti‐CD4 neutralizing antibody therapy. * indicates statistical significance at *p* < .05. In vivo experiments: *n* = 10.

Radiotherapy not only directly kills tumour cells but also transforms the tumour microenvironment from an immunosuppressive state to an immune‐activated state. The importance of TTN mutation in the context of tumour immunotherapy has been underscored by previous studies.^48^ Through prior bioinformatics analysis, we have found a substantial relationship between TTN mutation and tumour immune cell infiltration. Therefore, to investigate the impact of TTN on immune cells during radiotherapy, we initially employed CRISPR/Cas9 technology to knock out TTN in CT26 and MC38 cells, selecting the best TTN‐KO‐1 for subsequent experiments (Figure S7A). Subsequently, equal amounts of WT or TTN‐KO cells were subcutaneously inoculated into BALB/c or C57BL/6 mice, and tumour growth was monitored. Results revealed that in both the CT26 model in BALB/c mice and the immunocompetent C57BL/6 mouse model with MC38 cells, tumours with TTN‐KO exhibited decreased growth rates compared with the WT group, which further decreased in growth rate when combined with radiotherapy (Figure S7). Consequently, our findings suggest that the absence of TTN in mouse colon cancer cells inhibits tumour growth in vivo. Moreover, we conducted IHC staining and flow cytometry analysis to detect T‐cell infiltration. The results showed a noteworthy surge in the proportion of CD4^+^ and CD8^+^ T cells from the TTN‐KO cell group after radiotherapy as opposed to the WT group (Figure 7A–C, Figure S8A,B). The gating strategy used for flow cytometry immune cell analysis is illustrated in Figure S9.

Furthermore, to verify the contribution of CD4^+^ and CD8^+^ T cells to the anti‐tumour immune response post radiotherapy in the context of TTN, we selectively depleted these cell populations individually or simultaneously using corresponding antibodies in an MC38 cell line implanted model in C57BL/6 mice and analyzed their impact on tumour growth (Figure 7D). The outcomes illustrated that the reduction of CD4^+^ T and CD8^+^ T cells, or CD4/8^+^ T cells, weakened the inhibitory effect of TTN mutation on tumour growth post‐radiotherapy (Figure 7E). These results imply that the immune reaction against tumours triggered by TTN mutation may function through the infiltration of CD4^+^ and CD8^+^ T cells.

### 3.8 Immunomodulation mediated by TTN mutation and its association with radiotherapy outcomes in READ patients

**FIGURE 8:**
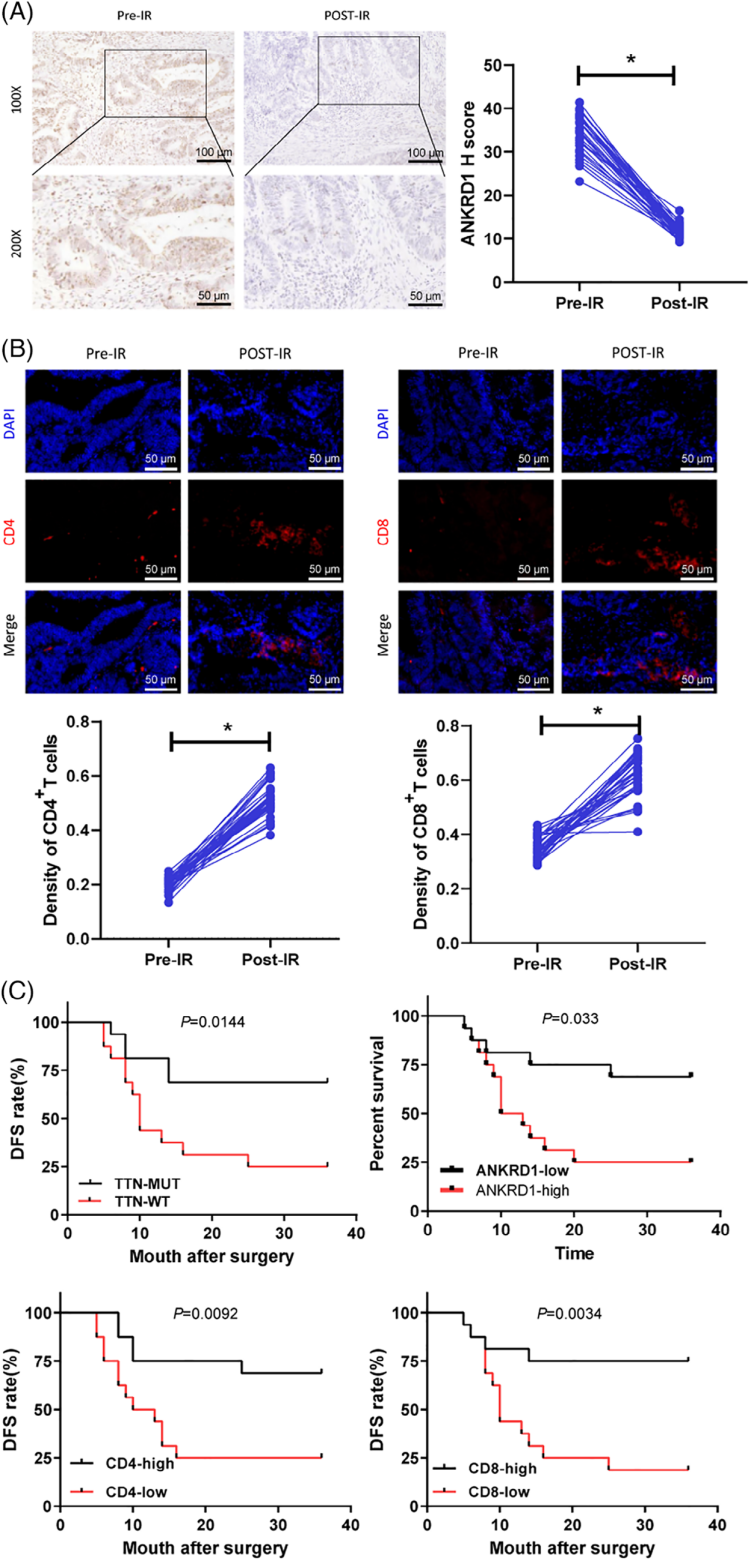
Immunogenic activation mediated by TTN mutation and its correlation with radiotherapy outcome in READ patients. (A) Immunohistochemical detection of post‐chemoradiotherapy positive expression of ANKRD1 (scale bar = 50 µm), *N* = 32. (B) Representative images of immunofluorescence staining of CD4 and CD8 (in red) in rectal tumour samples (scale bar = 50 µm). (C) Kaplan–Meier curve for DFS in READ radiotherapy patients. ***p < .001.

To assess the clinical impact of TTN mutation on the immune response during radiotherapy, paired tumour samples were obtained from 32 patients with READ who received a biopsy prior to radiotherapy and surgical resection later. Evaluation of ANKRD1 expression through IHC involved calculating the H‐score, revealing a significant decrease in ANKRD1 expression after radiotherapy (Figure 8A). Immunofluorescence staining also demonstrated a significant increase in CD4^+^ and CD8^+^ T cell numbers after radiotherapy (Figure 8B). Based on the ANKRD1 H‐score and the density of CD4 and CD8 positive staining, patients were divided into low and high groups. Additionally, patients were classified into wild‐type (WT) and mutant (MUT) TTN groups for Kaplan–Meier survival analysis. These analyses demonstrated that READ patients with TTN‐MUT, low ANKRD1 expression, and high density of CD4^+^ and CD8^+^ T cells had a longer 3‐year DFS (Figure 8C). The results indicate that the immune response activation triggered by TTN mutation influenced by radiotherapy has a beneficial effect on the prognosis of patients.

## 4 DISCUSSION

This study, for the first time, identifies TTN gene mutations as potential key genes affecting radiotherapy sensitivity and anti‐tumour treatment in READ patients. Through bioinformatics analysis and experimental validation, a substantial surge was noted in the sensitivity of READ cells with TTN mutations to radiotherapy, characterized by a decline in proliferation rate and an escalation in apoptosis post‐radiation exposure. While the role of the TTN gene in cardiomyopathy has been extensively studied, its function in cancer remains unclear.^14^, ^49^, ^50^ Our study offers fresh perspectives on the involvement of the TTN gene in cancer, indicating that TTN mutations may impact the radiotherapy sensitivity of READ cells.

TTN mutations may reduce the DDR, leading to increased DNA damage. This finding emphasizes the crucial role of TTN mutations in DNA damage repair processes.^12^, ^51^ Recent studies suggest that TTN mutations may influence cancer cell sensitivity to therapy through various mechanisms, including DNA repair and cell cycle regulation.^52^, ^53^, ^54^ These findings are consistent with our discoveries and further support the potential role of TTN mutations in cancer treatment.

**FIGURE 9:**
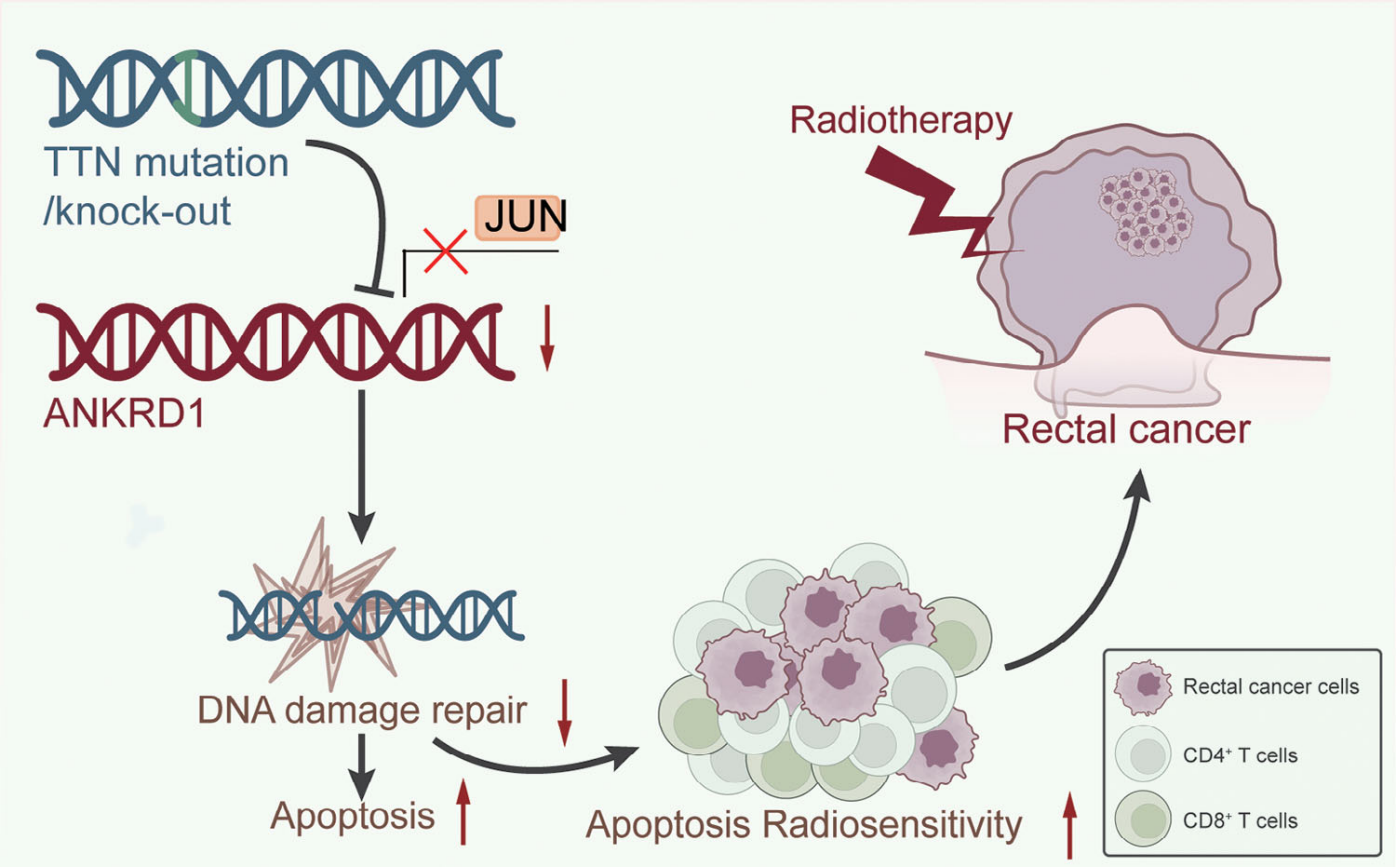
Schematic illustration of the molecular mechanism showing how TTN mutation inhibits ANKRD1 to alleviate DDR repair, thereby promoting radiotherapy sensitivity and activation of anti‐tumour immunity in READ.

Our research reveals, for the first time, an association between TTN mutations and ANKRD1. Specifically, we found that TTN mutations inhibit the expression of ANKRD1 by disrupting JUN recruitment. This finding aligns with previous studies showing that JUN can activate the transcription of ANKRD1. Research indicates that JUN enhances ANKRD1 expression directly through its transcriptional activity, further regulating crucial processes in heart development and pathological conditions.^55^ This mechanism affects cell cycle regulation and DNA damage repair capacity, slowing down the proliferation rate of READ cells, increasing apoptosis rates, and potentially enhancing radiotherapy sensitivity. Additionally, TTN mutations may promote the infiltration of CD4 and CD8 T cells, altering the TIME and boosting anti‐tumour immune activation (Figure 9). These results emphasize the crucial function of ANKRD1 in both heart disease and cancer, as shown in previous studies.^12^, ^51^ If further research confirms that TTN mutations indeed enhance the sensitivity of READ cells to radiotherapy, personalized radiotherapy plans tailored to patients’ TTN gene status could be developed in clinical practice.

Recent investigations have notably progressed our insights into the tumour immune microenvironment (TIM) and its role in influencing treatment responses in various cancer types. TIM's involvement in immune response regulation is widely acknowledged, particularly in enhancing the efficacy of immunotherapies and radiotherapy. For instance, it has been demonstrated that TIM composition, including the presence of immune‐suppressive cells, can influence the outcomes of anti‐cancer therapies.^56^ Additionally, the activation of CD8^+^ T cells in the TIM has been correlated with improved responses to radiotherapy and enhanced anti‐tumour immunity, as observed in multiple cancer types.^57^ Similarly, studies on immune cell infiltration have indicated that CD4^+^ and CD8^+^ T cells are key players in the modulation of tumour responses to radiation.^58^ Our findings are consistent with these observations, where TTN mutations were associated with increased CD4^+^ and CD8^+^ T‐cell infiltration, contributing to improved radiotherapy sensitivity in READ. These insights underscore the importance of targeting TIM components to improve cancer treatment outcomes.

Primarily based on bioinformatics analysis and laboratory models, although the results are promising, their efficacy and safety in human patients require further validation. Due to limitations in sample size, some findings need confirmation in larger sample populations and multicenter studies. Moreover, while TTN mutations may influence READ cell sensitivity to radiotherapy, the specific mechanisms necessitate further investigation.

As our understanding of the role of TTN mutations in radiotherapy for READ deepens, the development of personalized radiotherapy plans based on the patient's genetic status is within reach, potentially enhancing treatment outcomes. If TTN mutations do indeed augment anti‐tumour immunity by altering the tumour immune environment, TTN mutations could serve as new immunotherapy targets, paving the way for novel treatments for READ. Further research into the relationship between TTN mutations and ANKRD1 is anticipated to provide more insights into the aetiology of READ and facilitate the development of new therapeutic strategies.

## AUTHOR CONTRIBUTIONS

Z.J. and X.W. contributed equally as corresponding authors. H.L. and J.L. served as co‐first authors. H.L., J.L., and G.Y. conducted the experimental work and data analysis. X.G., Z.Z., P.C., and H.C. contributed to the bioinformatics analysis. Z.J. and X.W. supervised the study. All authors participated in the interpretation of results, manuscript writing, and final approval of the manuscript. Z.J. and X.W. provided critical revisions and guidance throughout the study. All authors reviewed and approved the final version of the manuscript for submission.

## CONFLICT OF INTEREST STATEMENT

The authors declare no conflict of interest.

## FUNDING

Not applicable.

## ETHICS STATEMENT

This study was approved by the Clinical Ethics Committee of the National Clinical Research Center of Cancer (no. NCC2016JZ‐06). All animal experiments were approved by the Animal Ethics Committee of the National Cancer Center (no. NCC2023A179).

## Supporting information



Supporting Information

## Data Availability

All data are available from the corresponding author upon a reasonable request.
