# Supplementary material for: Titin gene mutations enhance radiotherapy efficacy via modulation of tumour immune microenvironment in rectum adenocarcinoma
Source: Clin Transl Med. 2025 Jan 2;15(1):e70123. doi: 10.1002/ctm2.70123 (PMC11695211; doi:10.1002/ctm2.70123)
Supplement: Supplementary file 1 — Supporting Information [file CTM2-15-e70123-s001.docx]

**TTN Gene Mutations Enhance Radiotherapy Efficacy via Modulation of Tumor Immune Microenvironment in Rectum Adenocarcinoma**

**Running Title:** TTN Mutations in Rectal Cancer

**Hengchang Liu**^#^**, Jialiang Liu**^#^**, Guanhua Yu, Xu Guan, Zhixun Zhao, Pu Cheng, Haipeng Chen, Zheng Jiang^*^, Xishan Wang^*^**

Department of Colorectal Surgery, National Cancer Center/National Clinical Research Center of Cancer/Cancer Hospital, Chinese Academy of Medical Sciences and Peking Union Medical College, Beijing 100021, China.

^#^ These authors are regarded as co-first authors

**^*^** **Correspondence to:** **Zheng Jiang and Xishan Wang,** Department of Colorectal Surgery, National Cancer Center/National Clinical Research Center of Cancer/Cancer Hospital, Chinese Academy of Medical Sciences and Peking Union Medical College, No.17, Panjiayuan South Lane, Chaoyang District, Beijing 100021, China.

1. **mail**: [1705070229@xy.dlpu.edu.cn](mailto:1705070229@xy.dlpu.edu.cn) (**Zheng Jiang**); [1707040121@xy.dlpu.edu.cn](mailto:1707040121@xy.dlpu.edu.cn) (**Xishan Wang**)

**Tel.:** +86-18810817803/13552367779


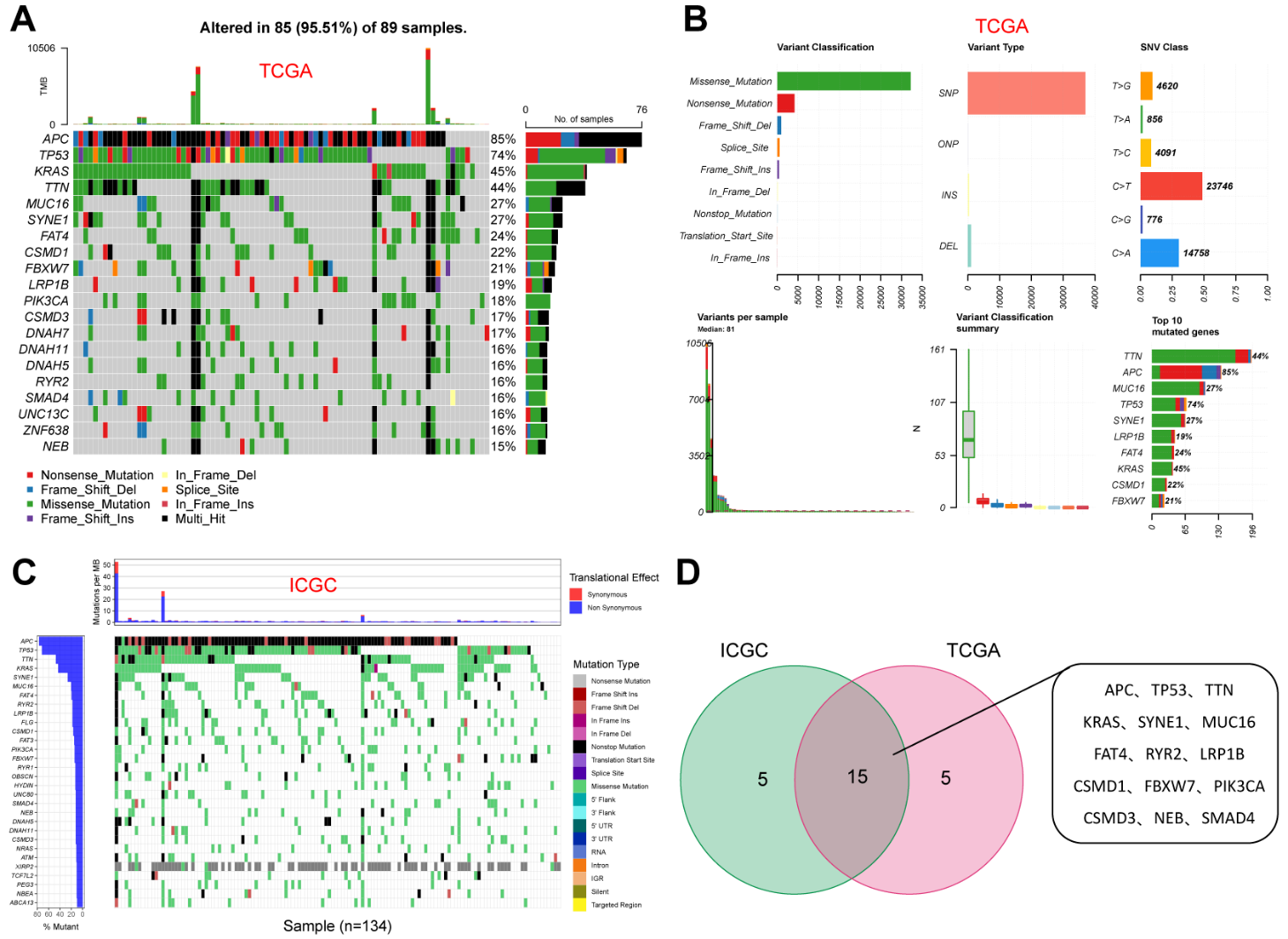


**Figure S1. Distribution of Frequently Mutated Genes in READ.**

Note: (A) Single nucleotide variant (SNV) data from the TCGA-READ (Rectum adenocarcinoma) dataset were downloaded from the TCGA website (<https://cancergenome.nih.gov/>). The "Masked Somatic Mutation" data were selected, displaying a waterfall plot of frequently mutated genes in TCGA-READ samples. The left panel shows the ranking of gene mutation frequencies, while the right panel presents the mutation frequencies, and different mutation types are depicted below, N=89. (B) Comprehensive overview of mutation classification, mutation types, and SNV classification in TCGA-READ samples. (C) Somatic gene mutation information for American READ samples was obtained from the ICGC database (<http://dcc.icgc.org/releases/current/Projects>) as of November 27, 2019, showing a waterfall plot of frequently mutated genes in ICGC-READ samples. The left panel displays the ranking of gene mutation frequencies, and the right panel shows different mutation types, N=134. (D) Venn diagram illustrating the overlap of frequently mutated genes covered by TCGA and ICGC datasets.


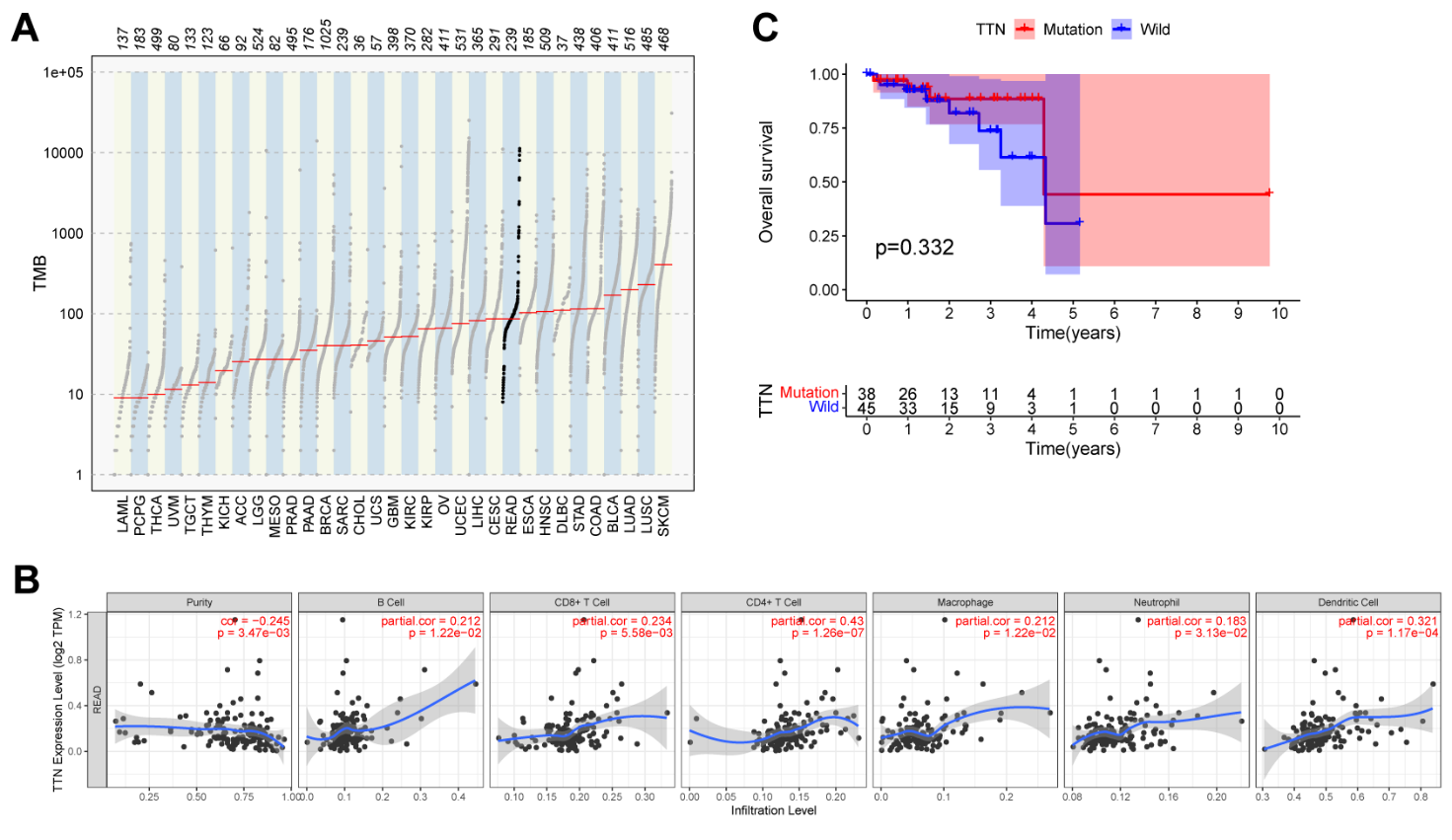


**Figure S2. Features of TTN Mutations.**

Note: (A) Pan-cancer TMB status in TCGA samples; (B) Correlation analysis of TTN gene expression levels with six immune cell types using TIMER database; (C) Overall survival analysis of TTN wild-type and mutant types in TCGA-READ samples.


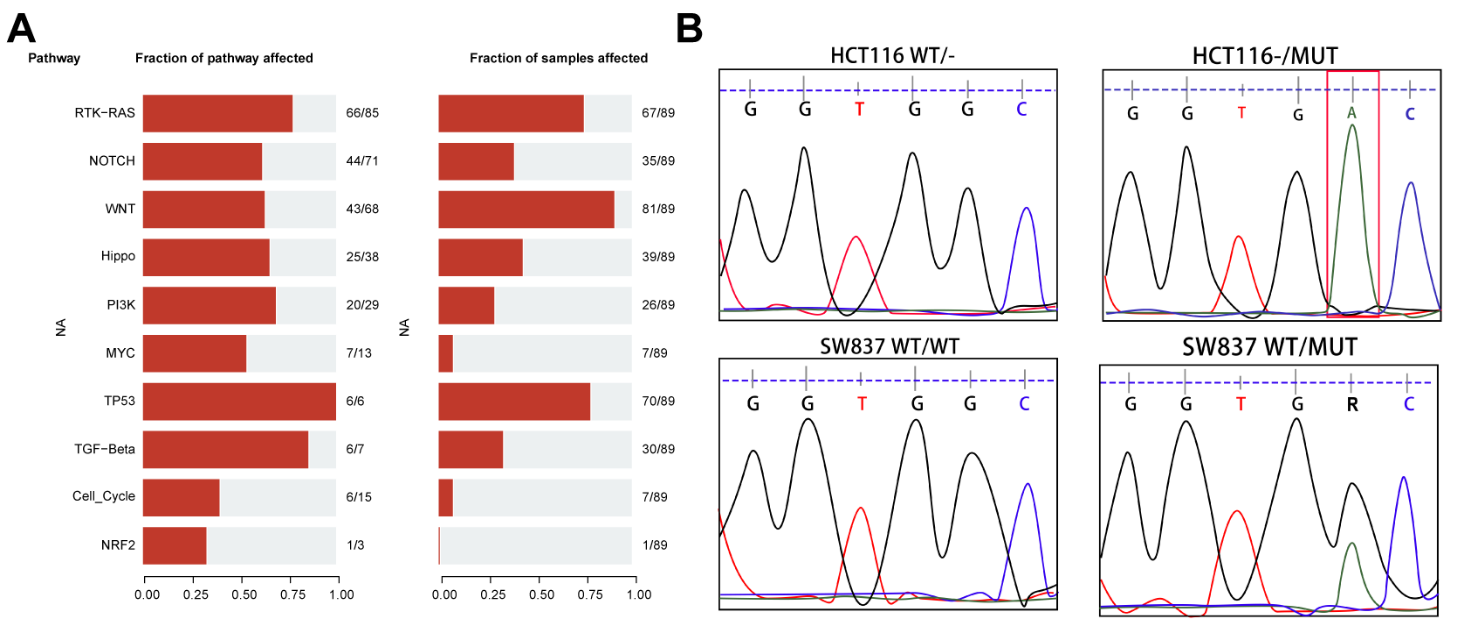


**Figure S3. Features of TTN Mutations.**

Note: (A) Enrichment analysis of gene mutation pathways in TCGA-READ samples; (B) Sanger sequencing confirmed the presence of TTN mutations in allele gene cell lines, such as HCT116 and SW837. The cell experiments were repeated three times, and 'R' indicates the detection of both G and A.


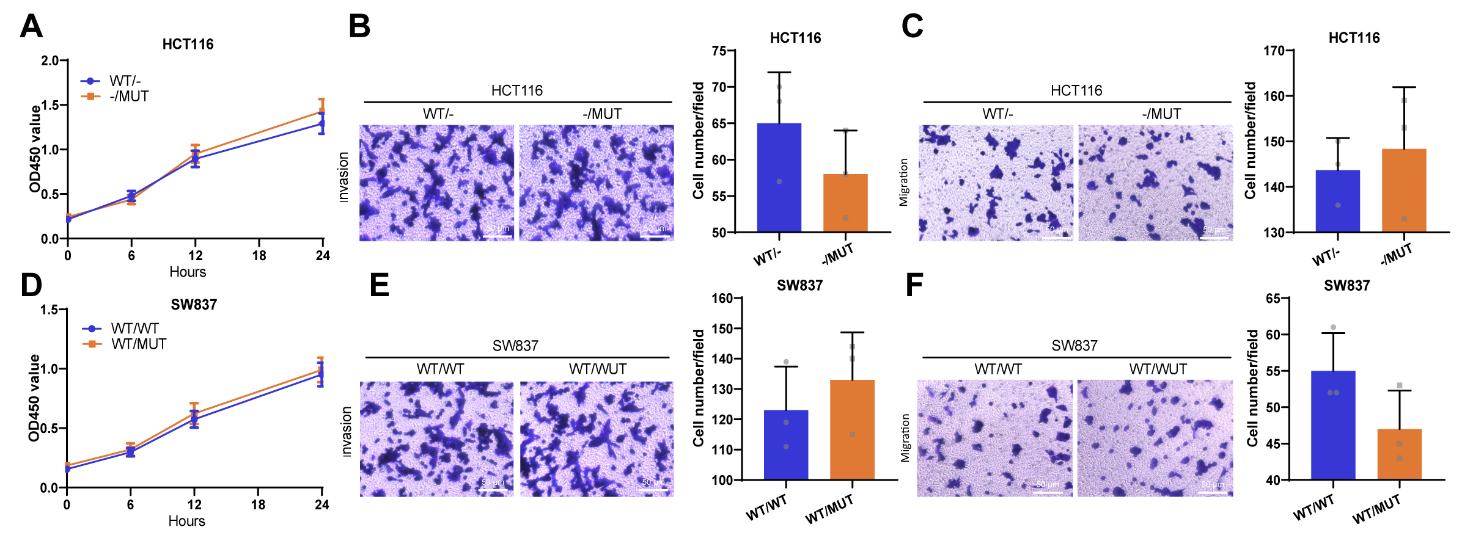


**Figure S4. The Impact of TTN Mutation on Cellular Biological Functions.**

Note: (A) The effect of TTN mutation on the proliferation of HCT116 cells; (B) Assessment of the influence of TTN mutation on the invasion of HCT116 cells using transwell assay; (C) Evaluation of the impact of TTN mutation on the migration of HCT116 cells using transwell assay; (D) Effects of TTN mutation on the proliferation of SW837 cells; (E) Assessment of the influence of TTN mutation on the invasion of SW837 cells using transwell assay; (F) Evaluation of the impact of TTN mutation on the migration of SW837 cells using transwell assay.


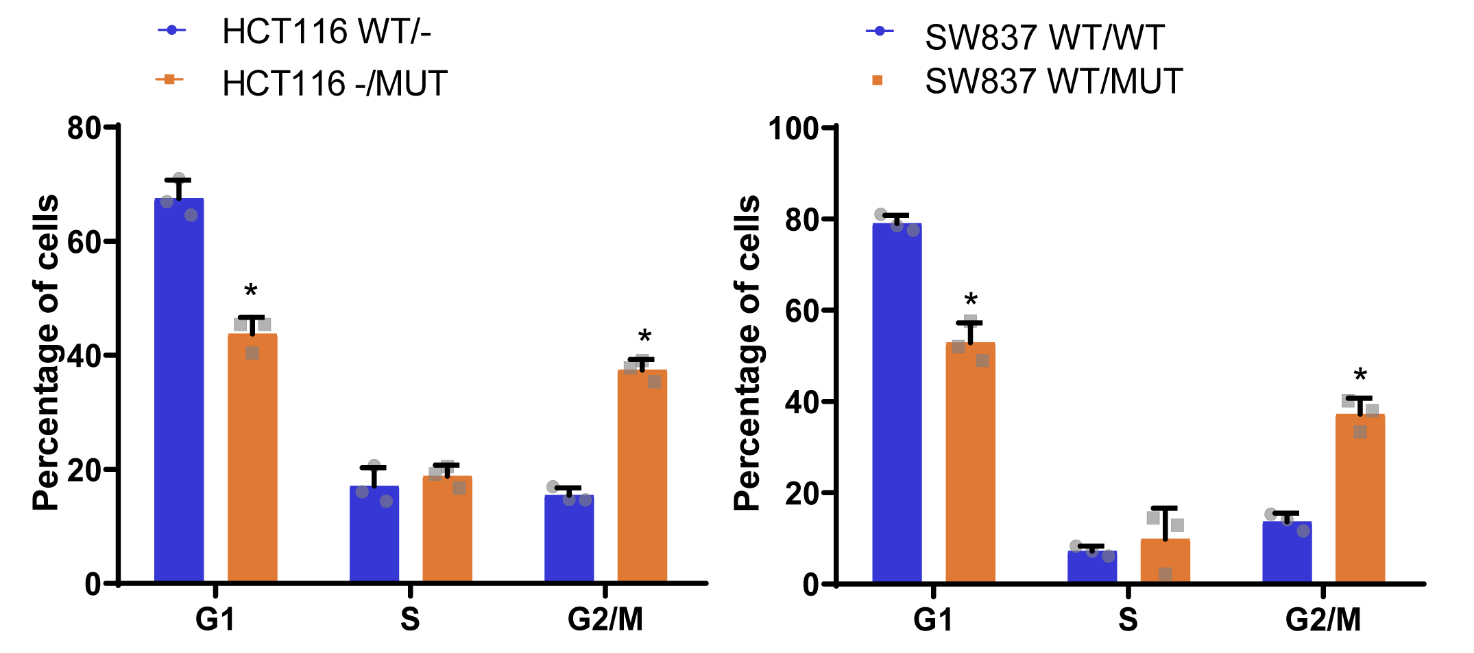


**Figure S5. Quantitative Analysis of the Cell Cycle in TTN Wild-Type and Mutant Gene Cells.**

Note: Flow cytometry was used to detect cell cycle distribution. * indicates P < 0.05. The cell experiments were repeated three times.


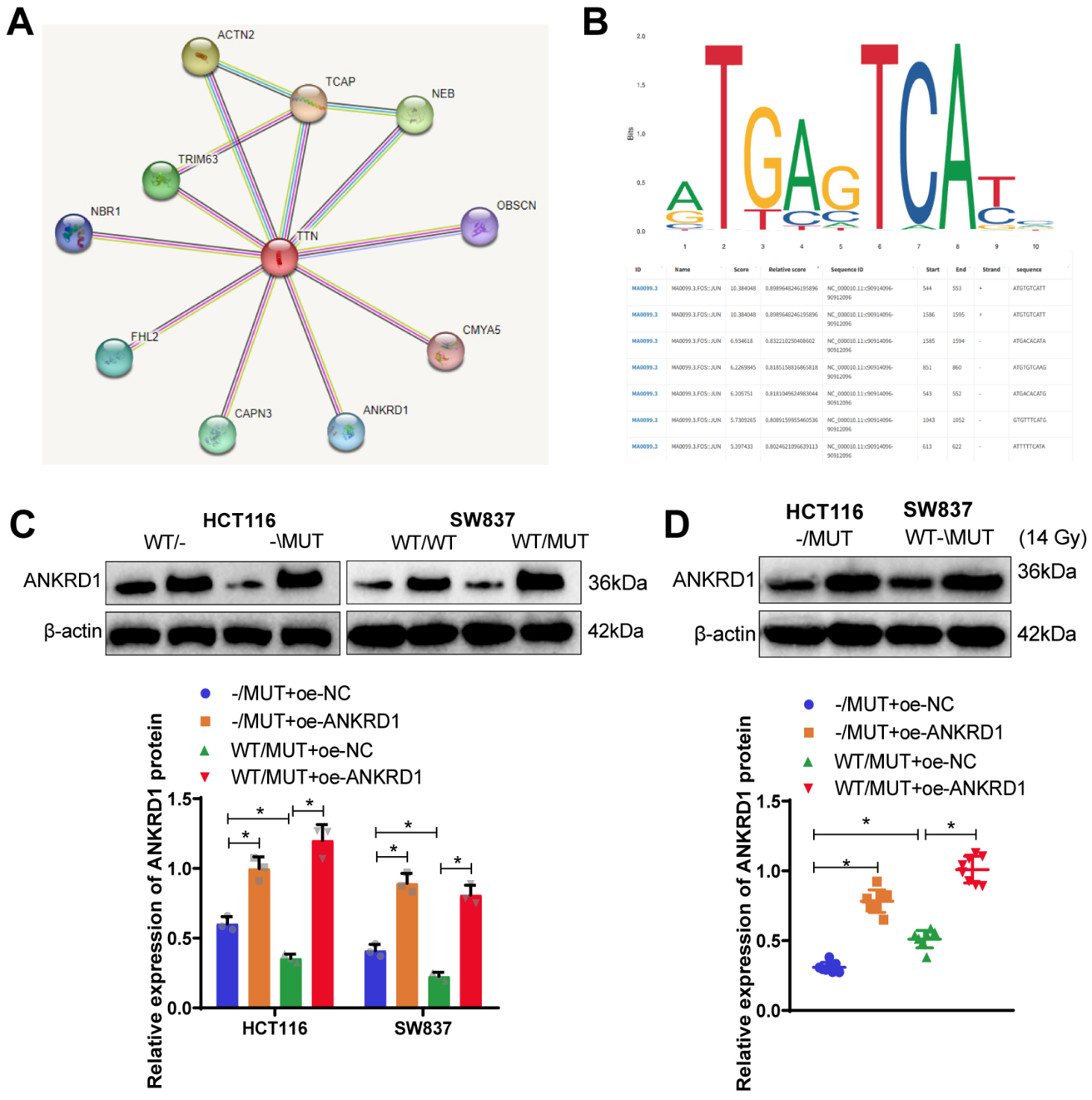


**Figure S6. ANKRD1 is a Gene Related to TTN Mutations.**

Note: (A) Protein network graph retrieved from STRING database (https://cn.string-db.org/cgi/input.pl) showing interactions with TTN, with a minimum interaction score of 0.9 as the filtering criterion; (B) Prediction of JUN binding site on ANKRD1 using JASPAR (https://jaspar.genereg.net); (C-D) Protein expression of ANKRD1 detected by Western blot in cells and tumor tissues. * indicates *P* < 0.05. Cell experiments were repeated three times, and the nude mice tumor formation experiment had a sample size of n=10.


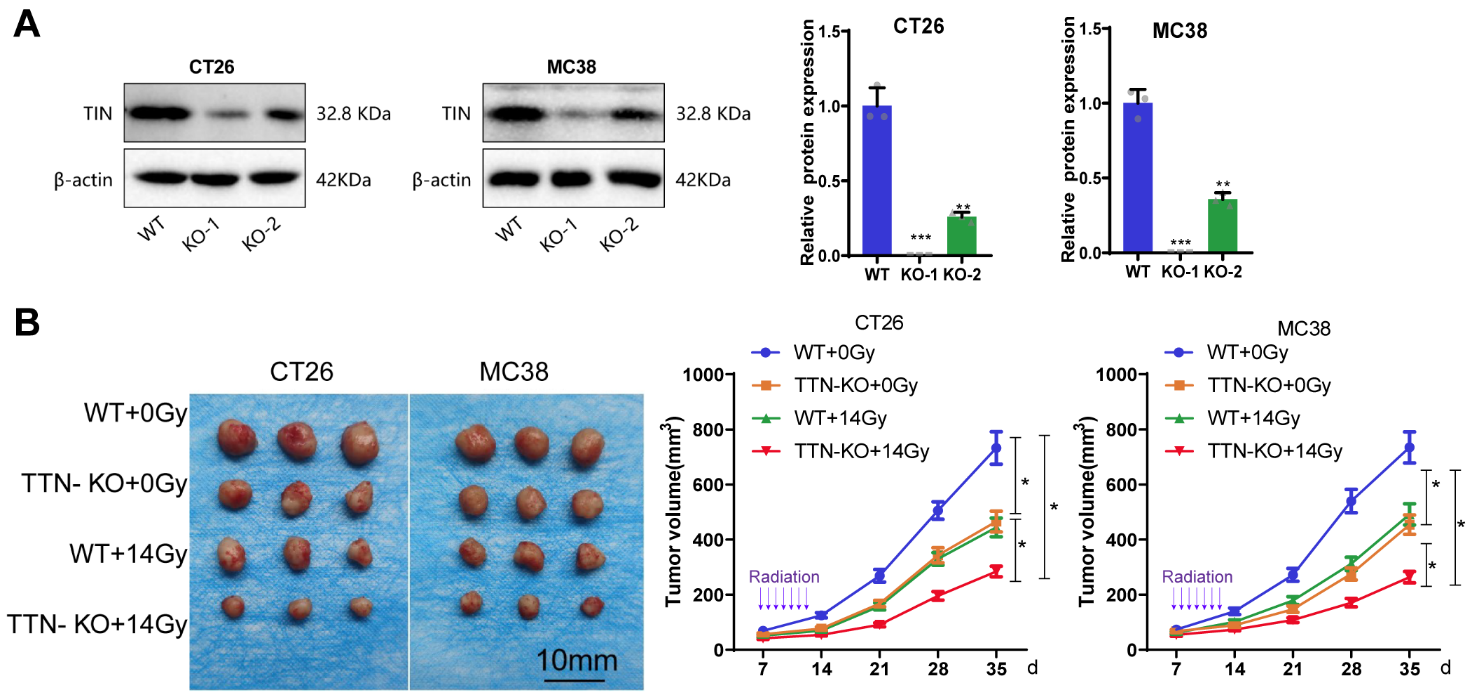


**Figure S7. Effects of TTN Knockout on Tumor Growth in Subcutaneous Transplantation Mouse Models After Radiotherapy.**

Note: (A) Assessment of TTN protein expression in CT26 and MC38 cells using Western Blot; (B) Changes in tumor volume of subcutaneous transplants in BALB/c mice with CT26 transplants and C57BL/6 mice with MC38 transplants. * indicates *P* < 0.05. *In vivo* experiments n=10.


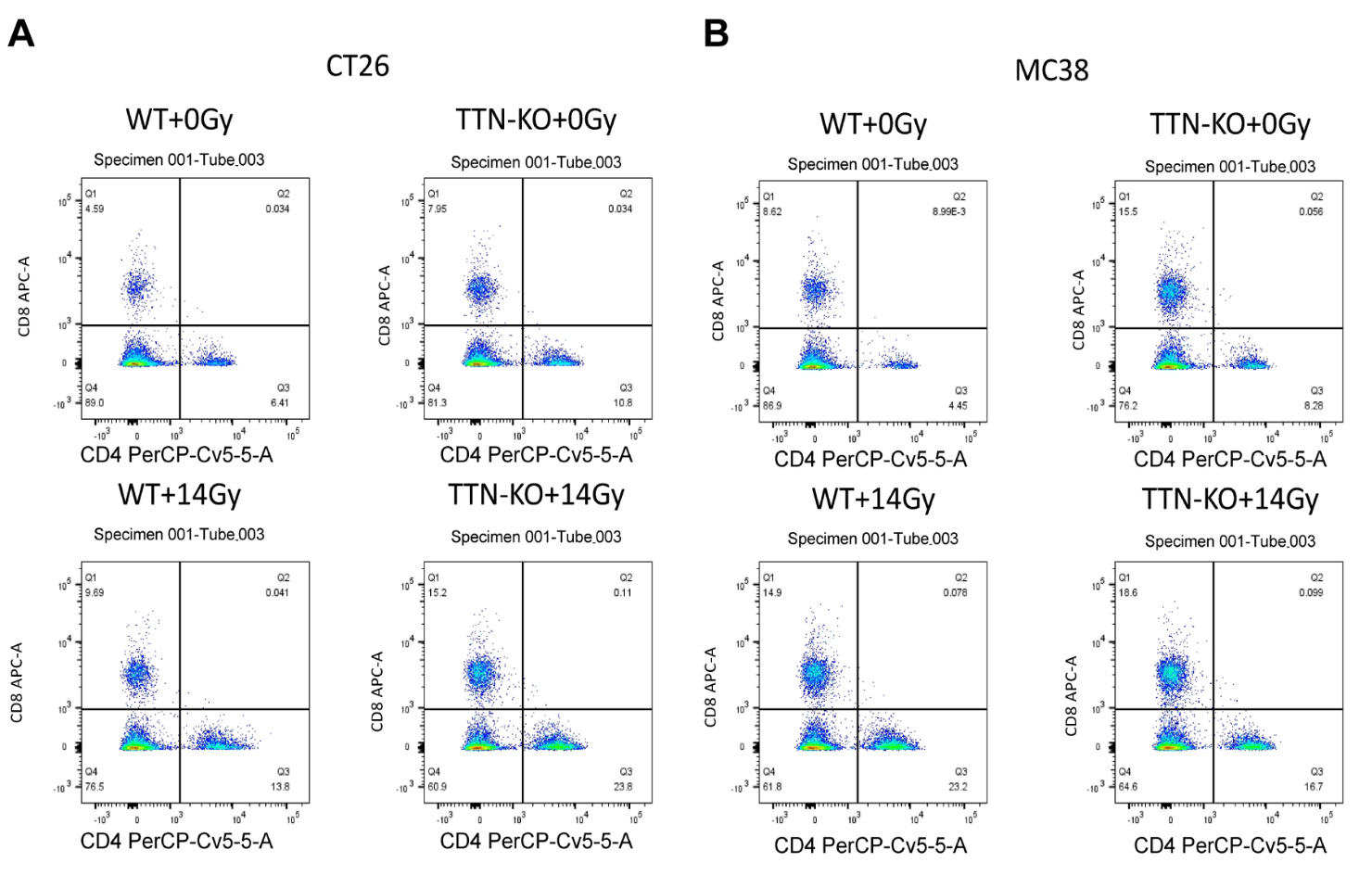


**Figure S8. Flow Cytometry Experiment Results.**

Note: (A) Flow cytometry analysis of CD4^+^ and CD8^+^ T lymphocyte proportions in tumor tissues from subcutaneously transplanted CT26 constructs in BALB/c mice; (B) Flow cytometry analysis of CD4^+^ and CD8^+^ T lymphocyte proportions in tumor tissues from subcutaneously transplanted MC38 constructs in C57BL/6 mice. The experiment was repeated three times.


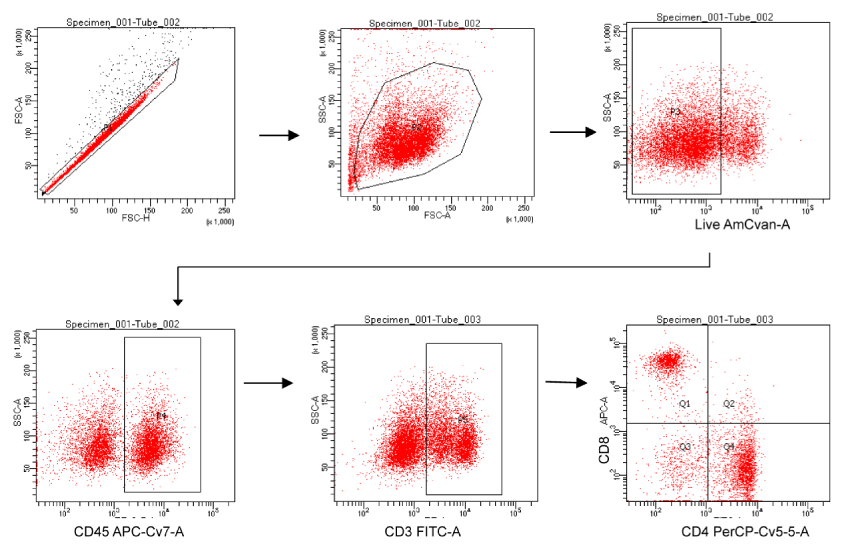


**Figure S9. Gate Strategy for Analysis of Splenic Immune Cells.**

Note: The methodology involved labeling live and dead cells with fixed active dyes and utilizing the following antibody combinations within the live cell gate to distinguish various immune cell populations: CD3^+^ leukocytes characterized as CD3^+^ CD45^+^ cells, CD8^+^ T cells as CD8^+^ CD3^+^ CD45^+^ cells, and CD4^+^ T cells as CD4^+^ CD3^+^ CD45^+^ cells. The experiment was repeated at least three times.

**Table S1. RT-qPCR primer sequence.**

| **Gene** | **Sequence (5'-3')** |
| --- | --- |
| TTN | F: CCCCATCGCCCATAAGACAC |
|  | R: CCACGTAGCCCTCTTGCTTC |
| ANKRD1 | F: GCCTACGTTTCTGAAGGCTG  R: GTGGATTCAAGCATATCACGGAA |
| GAPDH | F: CTGGGCTACACTGAGCACC |
|  | R: AAGTGGTCGTTGAGGGCAATG |

Note: F, forward; R. Reverse.
